# LCP2 mediates SUV39H1-driven cellular senescence-related chemoresistance in natural killer/T-cell lymphoma

**DOI:** 10.1038/s41419-026-08897-6

**Published:** 2026-05-28

**Authors:** Yue Zhang, Siyu Qian, Qing Yang, Zhuangzhuang Shi, Meng Dong, Zeyuan Wang, Zhenzhen Yang, Shaoxuan Wu, Zhaoming Li, Mingzhi Zhang, Xudong Zhang, Qingjiang Chen

**Affiliations:** 1 Department of Oncology, The First Affiliated Hospital of Zhengzhou University, Zhengzhou, China; 2 Henan Academy of Innovations in Medical Science, Zhengzhou, China

**Keywords:** Senescence, Oncogenes

## Abstract

Natural killer/T-cell lymphoma (NKTCL) is an aggressive haematological malignancy with poor prognosis, particularly in patients with relapsed/refractory (R/R) disease. The mechanisms underlying multidrug resistance in NKTCL remain unclear and present an urgent challenge that must be addressed during clinical treatment. Multidrug-resistant NKTCL models were established using adriamycin (ADM), and cellular senescence was confirmed by markers including P16, P21, and senescence-associated β-galactosidase (SA-β-gal). Proteomic sequencing of plasma from clinical patients and resistant cells identified LCP2 as a key protein. Phosphoproteomics, mass spectrometry, and co-immunoprecipitation analyses revealed LCP2’s role in mediating senescence-associated chemoresistance. An in vivo ageing microenvironment model was used to assess whether targeting the LCP2-mediated axis could eliminate chemoresistant senescent cells. Results show that ADM-resistant NKTCL cells exhibited phenotypic and senescence features. Of these, LCP2 expression was significantly reduced in the plasma of R/R NKTCL patients and in chemoresistant cells, correlating inversely with senescence marker SA-β-gal. Moreover, LCP2 knockdown enhanced the chemoresistance, senescent-associated secretory phenotype secretion, and G0/G1 cell cycle arrest in NKTCL cells. Mechanistically, LCP2 deficiency activated the IQGAP2/LaminA/C/SUV39H1 axis, thus driving DNA damage, telomere stress-induced senescence, and facilitating the formation of an immunosuppressive microenvironment. Importantly, targeting this axis with Epitalon and Chaetocin can partially eliminate therapy-induced senescent cells, enhance response to chemotherapeutics, and alleviate the immunosuppressive microenvironment to a certain extent in vivo. In conclusion, this study is the first to uncover LCP2 as a critical biomarker of senescence-related chemoresistance in NKTCL, providing a theoretical basis for the clinical translation of senolytics for treating R/R NKTCL.

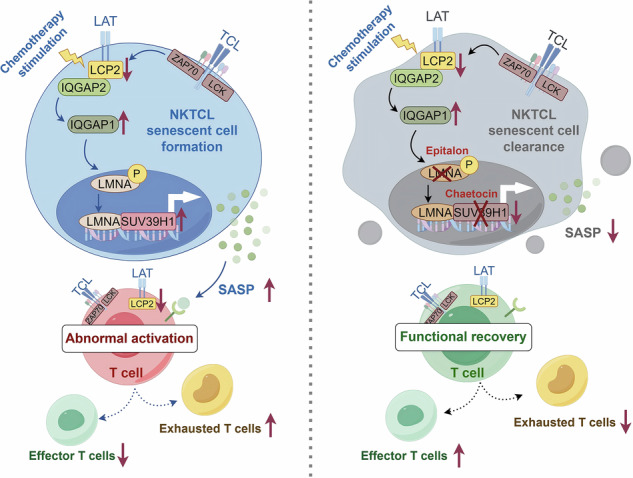

## Introduction

Natural killer (NK)/T-cell lymphoma (NKTCL) is an aggressive subtype of non-Hodgkin lymphoma associated with an unfavourable survival rate. It originates from NK and cytotoxic T cells and is strongly associated with Epstein-Barr virus (EBV) infection, with a geographical predilection for Latin American and Asian populations [1, 2].

Chemotherapy resistance is a major cause of treatment failure in NKTCL. The conventional chemotherapy regimen is an anthracycline-based CHOP (cyclophosphamide, adriamycin [ADM], vincristine sulphate, and prednisone) regimen. The 2-year overall survival (OS) of the CHOP regimen for the treatment of NKTCL is 40.5% [3] due to the high expression of the multidrug resistance gene MDR1 and its product, P-glycoprotein (P-gp) [4, 5]. In recent years, chemotherapy regimens, including DDGP (dexamethasone, cisplatin, gemcitabine, and pegaspargase), P-GEMOX (pegaspargase, gemcitabine, and oxaliplatin), and SMILE (methotrexate, dexamethasone, ifosfamide, L-asparaginase, and etoposide), have increased the 5-year OS to 74.3% [6]. However, even after receiving the CHOP regimen followed by salvage treatment with the DDGP regimen, patients still experience disease progression [7]. This suggests that ADM has multidrug resistance characteristics and that there are more complex resistance mechanisms in patients with NKTCL. Therefore, exploring the underlying mechanisms and strategies for overcoming chemotherapy resistance in NKTCL is important in clinical practice.

With the in-depth exploration of the tumour microenvironment, tumour resistance is no longer limited to the traditional mechanisms of resistance. Researchers have found that ADM-induced therapy-induced senescence (TIS) in lymphoma cells secretes a senescence-associated secretory phenotype (SASP) through autocrine and paracrine mechanisms to promote the reprogramming of neighbouring cells in the ageing microenvironment and acquire tumour stem cell-like properties to promote tumour progression [8–10]. Senescence-associated β-galactosidase (SA-β-gal) combined with SASP, including cytokines, growth factors, tumour necrosis factor, chemokines and matrix metalloproteinases, can serve as markers of the formation of senescent cells and ageing microenvironment [9, 11]. However, the association between drug-resistant NKTCL cells and senescent phenotypes has not yet been reported in existing studies. Additionally, senescence-related drug resistance biomarkers of NKTCL and their underlying mechanisms remain unknown.

Here, we demonstrate that chemoresistant NKTCL cells acquire phenotypic senescence features. We found that the T-cell receptor (TCR) adaptor protein lymphocyte cytosolic protein 2(LCP2), also known as SLP76, exhibited lower expression in NKTCL multidrug-resistant cells and in the plasma of relapsed or refractory (R/R) NKTCL patients than in patients with complete remission (CR). Mechanistically, we clarified that LCP2 deficiency activated the IQGAP2/LaminA/C/SUV39H1 axis, thus driving DNA damage, telomere stress-induced senescence, and facilitating the formation of an immunosuppressive microenvironment, providing new insights into the chemotherapy resistance mechanism of NK/T cell lymphoma.

## Materials and methods

### Cell lines and culture

NKYS and KHYG-1 cell lines were kindly provided by Dr. Wing C. Chan (City of Hope Medical Centre). SNK-6 cell line was kindly provided by Dr. Norio Shimizu of Chiba University. The SNT16 cell line was obtained from Guangzhou Bairui Biomedical Technology Co., Ltd. (Guangzhou, China). The RMA cell line was purchased from Shanghai WheLab Biotechnology Co., Ltd. (Shanghai, China). Resistance to adriamycin (ADM, Selleck, USA) was induced in the KHYG-1, NKYS, SNK-6, and SNT-16 cell lines by gradually increasing the ADM concentration in the culture medium, starting with an initial concentration of 50 ng/mL. The resistance index (RI) was calculated using the following formula: RI = IC50_(NKTCL/ADM)_/IC50_(NKTCL)_ [12]. Detailed culture conditions and procedures were provided in Supporting Information 1.

### Senescence-associated β-galactosidase (SA-β-gal) staining

Cells were collected by centrifuging into 1.5 mL centrifuge tubes, or frozen sections were attached to adhesive slides and then washed with PBS. The staining was performed according to the manufacturer’s instructions of the Senescence β-galactosidase Staining Kit (Beyotime, China). Detailed procedures were provided in Supporting Information 1.

### Establishment of the mouse model of drug-resistant ageing microenvironment

6–8 week-old F3 C57BL/6J-Lcp2^em1^ mice were used in this study and randomised into experimental groups. RMA cells were injected subcutaneously into the right flank of each mouse. Mice were euthanised in accordance with ethical guidelines. Detailed procedures were provided in Supporting Information 1.

### Statistical analysis

For comparisons between two groups, an unpaired Student’s t-test was applied. For comparisons among multiple groups, one-way ANOVA with Tukey’s post-hoc test was used. Detailed information was provided in Supporting Information 1.

## Results

### Chemotherapy-resistant NKTCL cell lines acquire phenotypic and functional senescence features

**Fig. 1:**
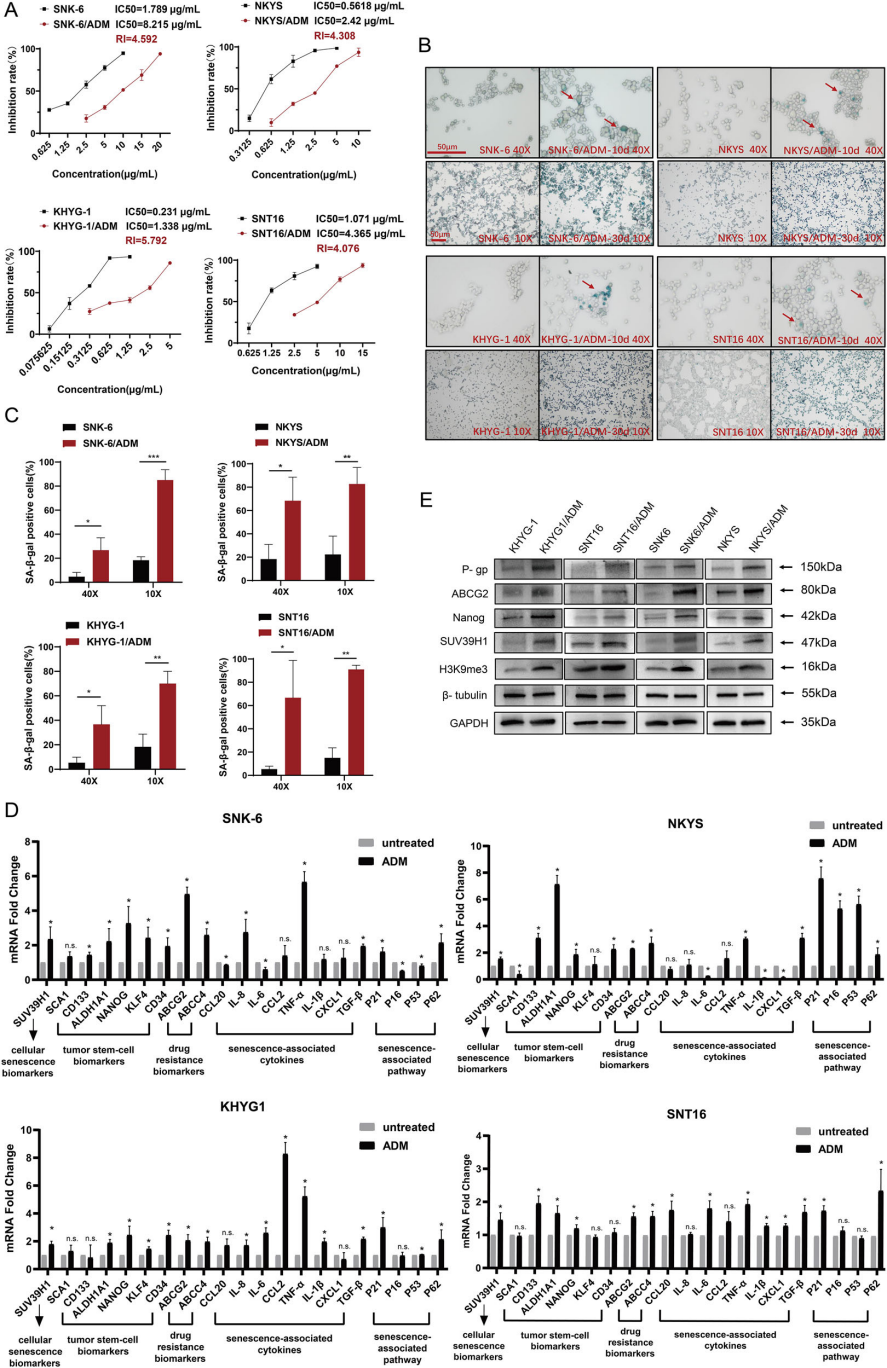
Chemotherapy-resistant NKTCL cell lines exhibit phenotypic and functional hallmarks of senescence. **A** Detection of ADM inhibition rate and RI in NKTCL drug-resistant cell lines and their parental cell lines (*n* = 3 per group); **B**, **C** β-galactosidase staining and statistical graphs of staining positivity rate in NKTCL drug-resistant cells and their parental cells (*n* = 3 per group); **D** Detection of mRNA expression of cellular senescence markers, drug resistance markers, cancer stem cell markers, senescence-related pathway genes, and representative SASP factors in NKTCL drug-resistant cells and their parental cells (*n* = 3 per group); **E** Detection of protein expression of cellular senescence markers, drug resistance markers, and cancer stem cell markers in NKTCL drug-resistant cells and their parental cells. *P < 0.05, **P < 0.01, ***P < 0.001, ns = no significance.

Because ADM can induce the establishment of multidrug-resistant cell lines [9, 12] and TIS [13, 14] phenotypes, we employed a low-dose ADM induction method to construct four NKTCL multidrug-resistant cell models, namely SNK-6/ADM, NKYS/ADM, KHYG-1/ADM, and SNT16/ADM, with all RI values greater than 4 (Fig. 1A). Then, we verified the senescence status of these four resistant cell lines using SA-β-gal staining. Senescent cells were first observed on day 10 of drug induction, and their numbers increased significantly by day 30 (Fig. 1B, C).

We selected relatively specific markers and genes for detection, including cellular senescence markers (SUV39H1) [15], cancer stem cell markers (SCA1, CD133, ALDH1A1, NANOG, KLF4, CD34), drug resistance markers (ABCG2, ABCC4), SASP factors (IL-1β, IL-6, IL-8, CCL-2, CCL-20, CXCL-1, TNF-α, TGF-β) [16], and senescence-related pathway genes (P16, P21, P53, P62) [17]. RT-qPCR was performed to measure the mRNA expression levels in resistant cells [9].

The results showed that compared with non-induced cells, the expression of the cellular senescence marker SUV39H1 was significantly upregulated in all four resistant cell lines (Fig. 1D). Additionally, the cancer stem cell markers ALDH1A1 and NANOG, drug resistance markers ABCG2 and ABCC4, SASP factors TNF-α and TGF-β, and senescence-related pathway genes P21 and P62 were all significantly upregulated in resistant cells (Fig. 1D). Consistent with these findings, Western blotting (WB) analysis further confirmed the increased protein expression of the drug resistance markers P-gp and ABCG2, stem cell marker Nanog, and cellular senescence markers H3K9me3 and SUV39H1 (Fig. 1E).

### LCP2 is a significant biomarker in TIS-mediated NKTCL chemoresistance

**Fig. 2:**
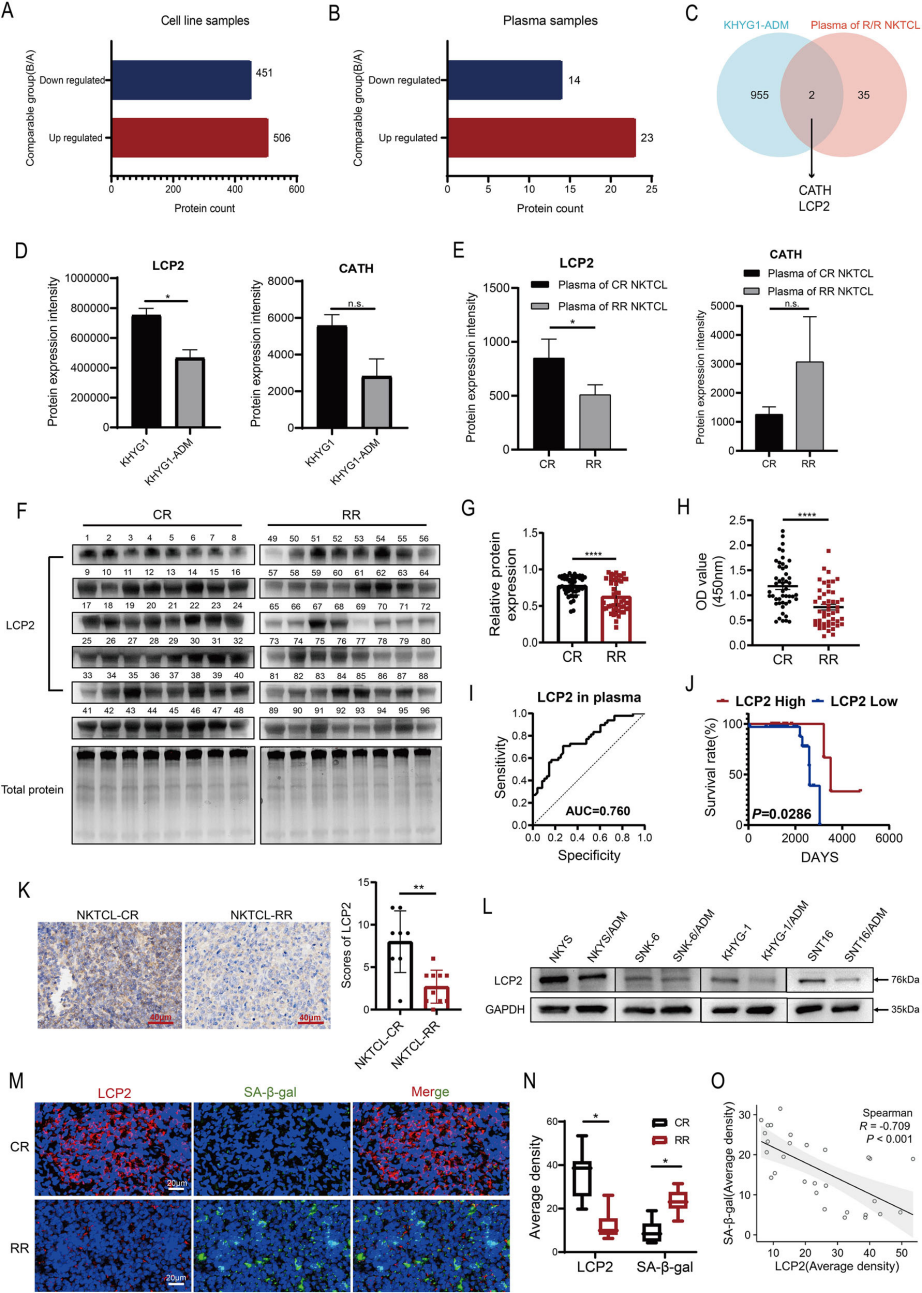
LCP2 emerges as a significant biomarker in TIS-related chemoresistance of NKTCL. **A** Differentially expressed proteins from TMT proteomics analysis of KHYG1-ADM and KHYG1 cell line samples (*n* = 3 per group); **B** Differentially expressed proteins from blood-DIA quantitative proteomics analysis of NKTCL plasma samples (*n* = 5 per group); **C** Venn diagram showing overlapping proteins between cell line and plasma proteomics data from (**A**, **B**). **D**, **E** Protein expression intensities of LCP2 and CATH in cells and plasma from proteomics analysis; **F**, **G** WB detection of LCP2 protein expression in plasma from patients with NKTCL with CR/RR, and bar graph showing relative protein expression statistics (*n* = 48 per group); **H** ELISA detection of LCP2 protein expression in plasma from patients with NKTCL with CR/RR (*n* = 48 per group); **I** ROC curve for determining the cut-off value of LCP2 expression; **J** Survival rates of the LCP2 high-expression group (*n* = 14) and LCP2 low-expression group (*n* = 34) in plasma from NKTCL patients; **K** LCP2 expression in tissues from CR/RR NKTCL patients detected using IHC, and bar graph showing LCP2 expression scores (CR, *n* = 8; RR, *n* = 10); **L** Comparison of LCP2 expression between NKTCL cell lines and NKTCL-ADM cell lines; **M**, **N** Co-localisation of LCP2 and SA-β-gal in NKTCL patient tissues, and bar graph showing fluorescence intensity statistics (CR, *n* = 15; RR, *n* = 14); **O** Correlation analysis of average fluorescence density between LCP2 and SA-β-gal. Scale bar = 20 μm. *P < 0.05, **P < 0.01, ****P < 0.0001, n.s. = no significance.

We selected the KHYG-1/ADM and KHYG1 cell lines with the highest RI for TMT-based proteomic sequencing and identified 506 upregulated and 451 downregulated proteins (Fig. 2A). Peripheral blood plasma samples were collected from patients with NKTCL in CR and from the same patients during the R/R phase. These samples underwent blood-DIA proteomic sequencing, which revealed 23 upregulated and 14 downregulated proteins (Fig. 2B). A comparison of differentially expressed proteins between the cellular and plasma datasets was performed, and Venn diagram analysis identified two commonly expressed proteins, CATH and LCP2 (Fig. 2C). Further analysis of protein expression intensity showed no statistically significant difference in CATH expression, whereas LCP2, which was downregulated, exhibited a statistically significant difference (Fig. 2D, E). Therefore, LCP2 was selected for the subsequent investigations.

LCP2 functions as a signalling adaptor protein and plays a key role in regulating the assembly of TCR signalling complexes and phosphorylation of downstream molecules [18]. In the present study, we validated LCP2 expression in NKTCL plasma, tissue, and drug-resistant cell models. First, WB and ELISA analyses were performed to detect LCP2 protein expression in plasma samples from additional patients. The results showed that LCP2 expression was downregulated in the plasma of R/R patients compared to that in CR patients (Fig. 2F–H). ROC analysis determined a cutoff value of 0.918 (OD value) for classifying high and low LCP2 expression using ELISA. Moreover, in the R/R group, patients with low LCP2 expression had a significantly shorter prognostic survival than those with high LCP2 expression (Fig. 2I, J). Second, IHC staining was used to validate LCP2 expression in tissue samples from patients with CR and R/R NKTCL, revealing downregulated LCP2 expression in R/R NKTCL tissues (Fig. 2K). Third, LCP2 expression was detected in normal NK/T cells, NKTCL cell lines, and NKTCL drug-resistant cell lines, showing significant downregulation in resistant cells compared to untreated cells (Fig. 2L and S1). To further explore the association between LCP2 and TIS, immunofluorescence assays were performed to examine the co-expression of LCP2 and the cellular senescence marker SA-β-gal in NKTCL tissues from CR and R/R patients. Compared to CR tissues, R/R tissues showed downregulated LCP2 expression and upregulated SA-β-gal expression (Fig. 2M, N), with a significant negative correlation between the two markers (Fig. 2O).

### Downregulated-LCP2 promotes NKTCL senescence-associated chemoresistance

**Fig. 3:**
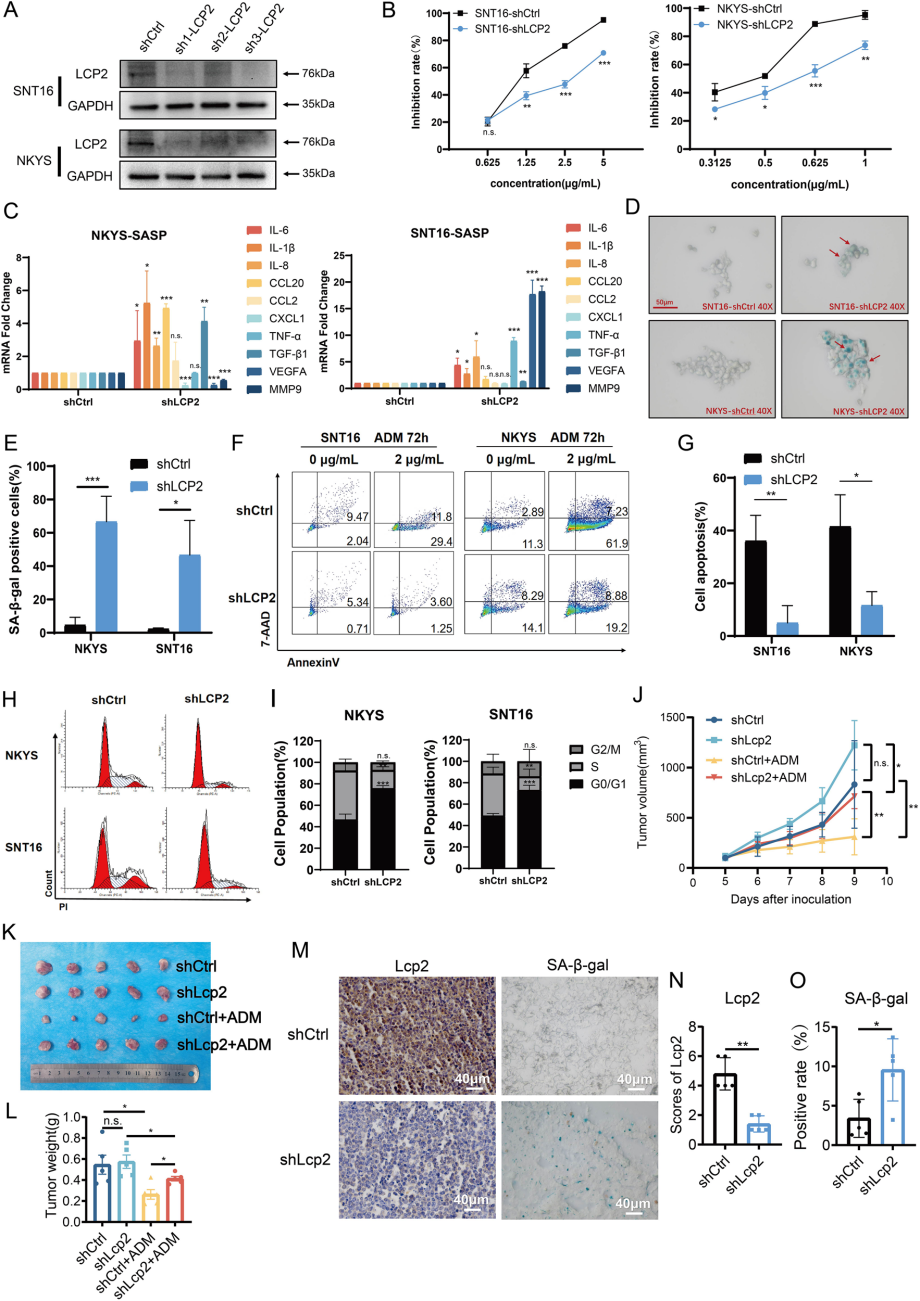
LCP2 downregulation facilitates senescence-related chemoresistance in NKTCL. **A** Construction of NKTCL-shLCP2 and NKTCL-shCtrl cell lines; **B** Comparison of ADM inhibition rates between NKTCL-shLCP2 and NKTCL-shCtrl cell lines (*n* = 3 per group); **C** RT-qPCR detection of SASP secretion in NKTCL-shLCP2 and NKTCL-shCtrl cell lines (*n* = 3 per group); **D**, **E** β-galactosidase staining for comparison of cellular senescence between NKTCL-shLCP2 and NKTCL-shCtrl cell lines, and bar graph showing SA-β-gal positive rate statistics (*n* = 3 per group); **F**, **G** Flow cytometry analysis of apoptosis in ADM-treated NKTCL-shLCP2 and NKTCL-shCtrl cell lines, with the quantitative bar graph (*n* = 3 per group); **H**, **I** Flow cytometry analysis of cell cycle arrest and the corresponding statistical bar graph showing cell cycle distribution in NKTCL-shLCP2 and NKTCL-shCtrl cell lines (*n* = 3 per group); **J** Tumour volume growth curves (*n* = 5 per group); **K** Comparison of tumour sizes among the four groups (*n* = 5 per group); **L** Comparison of tumour weights among the four groups (*n* = 5 per group); **M**–**O** Comparison of Lcp2 and SA-β-gal expression in tumours from the shLcp2+ADM and shCtrl+ADM groups (*n* = 5 per group). *P < 0.05, **P < 0.01, ***P < 0.001, ns = no significance. ADM adriamycin.

To investigate the biological functions of LCP2 in cellular senescence and chemoresistance, we generated NKTCL cell lines by LCP2 knockdown (shLCP2). Based on the expression levels of LCP2 in different NKTCL cell lines (Fig. S1), we selected the NKYS and SNT16 cell lines, which have relatively high LCP2 expression, for knockdown experiments. The sh3 plasmid, which showed the highest knockdown efficiency, was selected for subsequent experiments (Fig. 3A). The drug resistance of the shLCP2 cells were evaluated using the CCK-8 assay. Compared to shCtrl cells, the inhibition rate of shLCP2 cells treated with ADM was significantly reduced (Fig. 3B). A similar trend was observed following treatment with gemcitabine, cisplatin, oxaliplatin, L-asparaginase, and bendamustine (Fig. S6A–E). RT-qPCR was performed to detect the expression of SASP factors in shLCP2 cells, revealing that the levels of IL-6, IL-1β, and IL-8 were significantly increased in both cell lines (Fig. 3C). SA-β-gal staining was used to compare cellular senescence, and the results showed that the senescence rate of shLCP2 cells was significantly higher than that of shCtrl cells (Fig. 3D, E). Flow cytometry was used to assess the apoptotic effects of ADM on shLCP2 and shCtrl cells. After treatment with 2 μg/mL ADM for 72 h, the apoptosis rate of shLCP2 cells was significantly lower than that of shCtrl cells (Fig. 3F, G). Similarly, flow cytometric analysis of the cell cycle showed that a higher proportion of shLCP2 cells were arrested in the G0/G1 phase than shCtrl cells (Fig. 3H, I).

Subsequently, stable RMA-shLcp2 and RMA-shCtrl cell lines were established using the syngeneic NKTCL cell line RMA (Fig. S2A). These cells were subcutaneously implanted into C57BL/6 J mice to generate a tumour-bearing mouse model. Tumour volume growth curves were plotted and tumour weights were compared, showing no significant difference in tumour growth between the shLcp2 and shCtrl groups (Fig. 3J–L). However, after ADM treatment (administered from day 5 to day 9) in the shCtrl+ADM and shLcp2+ADM groups, the shLcp2+ADM group exhibited significantly greater chemoresistance compared with the shCtrl+ADM group (Fig. 3J–L). HE staining and immunohistochemical analysis of the excised tumour tissues confirmed that the tissues displayed NKTCL morphology and expressed markers, such as cytoplasmic CD3, CD56, Granzyme B, and TIA1 (Fig. S2B). Compared with tumour tissues in the shCtrl group, those in the shLcp2 group showed significantly downregulated Lcp2 protein expression and upregulated SA-β-gal activity (Fig. 3M–O).

Moreover, to explore a more robust causal relationship between LCP2 downregulation and senescence-associated chemoresistance, we used SNT16-ADM and NKYS-ADM cell lines to construct LCP2-overexpressing cell lines and their corresponding control cell lines (NKTCL-ADM-LvLCP2 and NKTCL-ADM-LvCtrl cells). Comparing NKTCL-ADM and NKTCL-ADM-LvCtrl cells can be used to exclude the effect of the plasmid vector on LCP2 expression. Subsequently, the overexpression of LCP2 protein in SNT16-ADM and NKYS-ADM cells was confirmed by WB (Fig. S3A). Further experiments using WB and SA-β-gal staining confirmed that compared with NKTCL-ADM-LvCtrl, the expressions of cellular senescence markers P16, P21, and SA-β-gal in NKTCL-ADM-LvLCP2 were significantly decreased (Fig. S3A–D). The cell apoptosis rate and cell viability after ADM treatment were detected by flow cytometry and CCK-8 assay, respectively, which confirmed that compared with NKTCL-ADM-LvCtrl, NKTCL-ADM-LvLCP2 cells exhibited enhanced sensitivity to ADM (Fig. S3E–G).

To investigate the effect of low LCP2 expression on cellular senescence in normal T cells and explore the ageing microenvironment of NKTCL further, we knocked down LCP2 in primary T cells and performed SA-β-gal staining as well as RT-qPCR assays. We found that LCP2 knockdown significantly promoted both cellular senescence and secretion of SASP factors in these cells (Fig. S4A, B).

### LCP2 mediates NKTCL senescence-associated chemoresistance via IQGAP2/LaminA/C/SUV39H1 axis

**Fig. 4:**
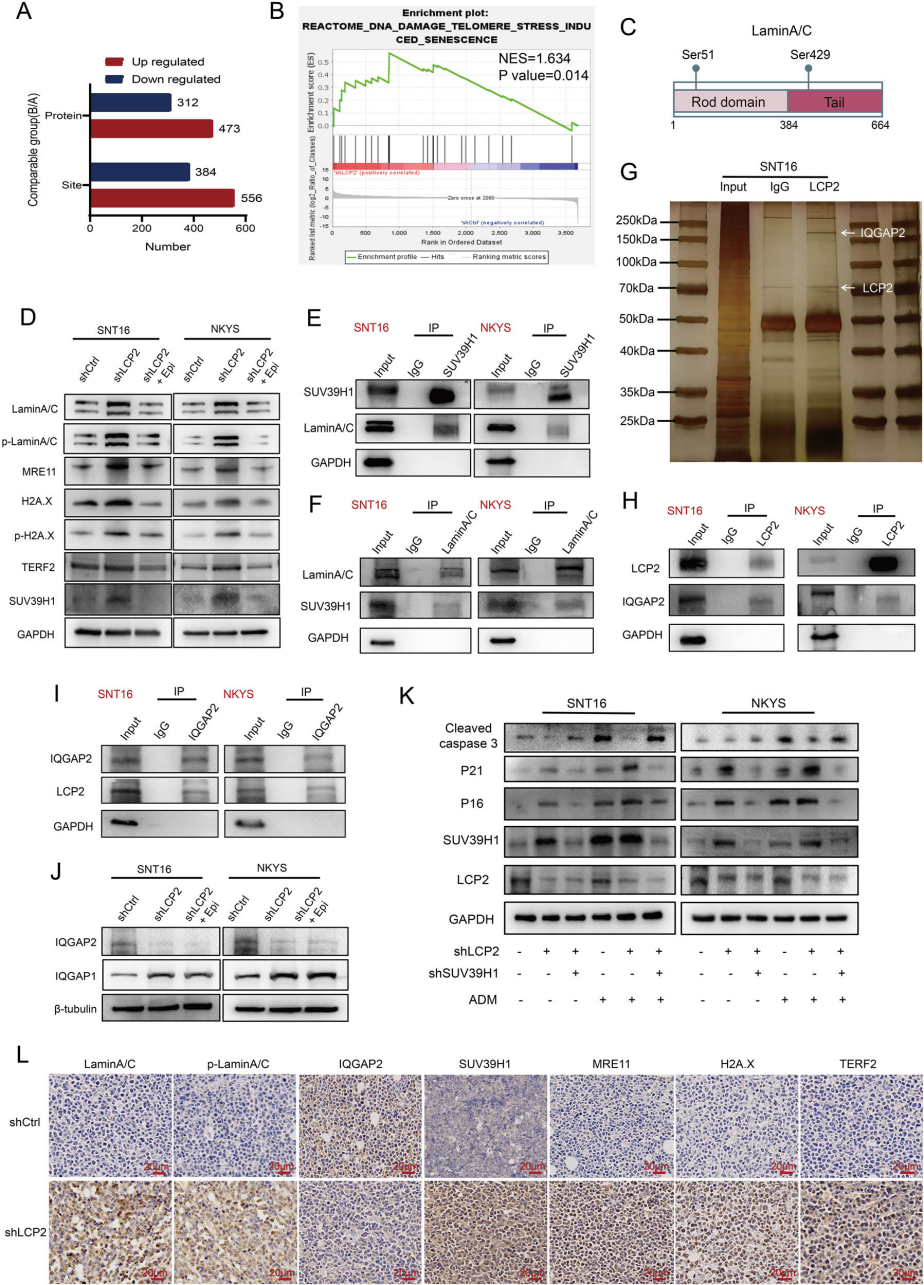
LCP2 contributes to senescence-related chemoresistance in NKTCL via the IQGAP2/LaminA/C/SUV39H1 signalling axis. **A** Phosphorylation sites with differential expression identified by phosphoproteomic sequencing in SNT16-shCtrl and SNT16-shLCP2 cell lines. **B** Phosphoproteomic enrichment analysis revealed significant upregulation of the DNA damage telomere stress-induced senescence pathway. **C** Significantly increased phosphorylation at Ser51 and Ser429 sites of LaminA/C within the identified regulatory network. **D** Relative protein expression detected using WB following inhibition of the DNA damage telomere stress-induced senescence pathway. **E**, **F** Protein-protein interaction between SUV39H1 and LaminA/C detected using the co-IP assay. **G** Interacting partners of LCP2 identified using silver staining assay. **H**, **I** Protein interaction between LCP2 and IQGAP2 detected using a co-IP assay. **J** Expression of IQGAPs protein in shCtrl, shLCP2, and Epitalon-treated shLCP2 cell lines. **K** Effects of LCP2 and SUV39H1 knockdown on chemoresistance and senescence phenotypes assessed by rescue experiments. **L** Representative IHC staining of senescence-related proteins in RMA-shLcp2 and RMA-shCtrl tumour tissues (200×).

To further investigate the cascade of molecular reactions and post-translational modifications in the cytoplasm triggered by decreased LCP2 expression, we performed 4D-Fast DIA quantitative phosphoproteomic analysis of SNT16 shCtrl and shLCP2 cells. Our analysis identified 473 upregulated proteins, 312 downregulated proteins, 556 upregulated phosphorylation sites, and 384 downregulated phosphorylation sites (Fig. 4A). Enrichment analysis revealed that multiple upregulated proteins following LCP2 knockdown were enriched in the “DNA damage telomere stress-induced senescence” regulatory network (Fig. 4B). Among these, the phosphorylation levels of Lamin A/C at Ser51 and Ser429 significantly increased (Fig. 4C). To validate the activation of this senescence-associated regulatory network, we examined the expression of key proteins (Lamin A/C, p-Lamin A/C, MRE11, H2A.X, p-H2A.X, TERF2, and SUV39H1) in the presence or absence of Epitalon, an anti-senescence drug. The results showed that shLCP2 cells exhibited higher expression of key proteins in the DNA damage/telomere stress-induced senescence network than shCtrl cells, and the Epitalon effectively inhibited the activation of this network (Fig. 4D).

To investigate how “DNA damage telomere stress-induced senescence” affect the ageing microenvironment, we focused on SUV39H1, a known LaminA/C interactor [19] and a key driver of senescence-associated drug resistance in lymphoma cells [9, 20]. Co-IP assays confirmed the interaction between LaminA/C and SUV39H1 (Fig. 4E, F). Considering that LCP2 is expressed in the cytoplasm, while SUV39H1 is expressed in the nucleus, we further explored the molecular signalling relationship between these two molecules. Silver staining combined with mass spectrometry identified IQGAP2 as a protein that directly interacts with LCP2, which was validated by Co-IP (Fig. 4G–I).

Notably, IQGAP2 expression decreased with LCP2 knockdown, thereby releasing the scaffold protein IQGAP1, which in turn promoted high expression of nuclear envelope LaminA/C and its phosphorylated sites. Importantly, IQGAP2 and IQGAP1 expression were not inhibited by Epitalon, confirming their roles as upstream regulators of the senescence-associated regulatory network (Fig. 4J). Rescue experiments confirmed that low LCP2 expression upregulated SUV39H1, which increased senescence markers P16 and P21 while reducing cleaved caspase 3 levels. This demonstrates that either low LCP2 or SUV39H1 overexpression enhances chemoresistance. (Fig. 4K).

IHC assays further validated that LCP2 mediates NKTCL senescence-associated chemoresistance through the IQGAP2/LaminA/C/SUV39H1 axis (Fig. 4L).

### SUV39H1 drives cellular senescence and mediates abnormal differentiation of T cells in ageing microenvironment

**Fig. 5:**
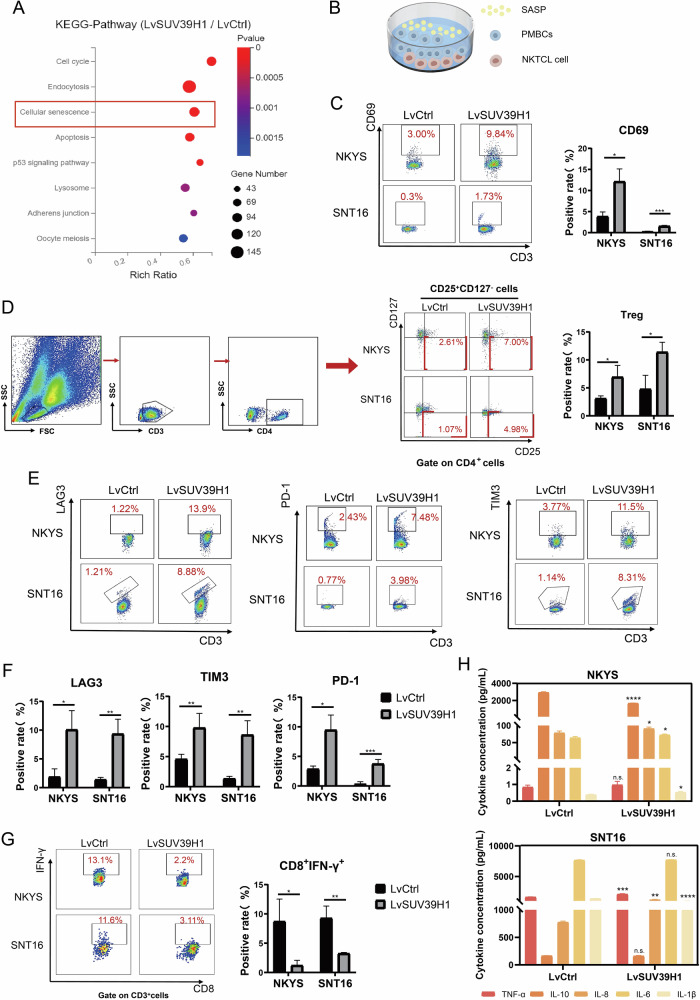
SUV39H1 regulates the differentiation of effector T cells and exhausted T cell subsets. **A** KEGG enrichment analysis of RNA-seq data between SNT16-LvCtrl and SNT16-LvSUV39H1 cells (*n* = 3 per group); **B** Schematic diagram of co-culture system between NKTCL cells and PBMCs; **C** Comparison of the proportion of activated T cells (CD3^+^CD69^+^ T cells) between the LvSUV39H1 group and LvCtrl group (*n* = 3 per group); **D** Comparison of the proportion of regulatory T cells (CD4^+^CD25^+^CD127^-^ T cells) between the LvSUV39H1 group and LvCtrl group (*n* = 3 per group); **E**, **F** Comparison of the proportions of exhausted T cells (CD3^+^PD-1^+^ T cells, CD3^+^LAG3^+^ T cells, CD3^+^TIM3^+^ T cells) between the two groups (*n* = 3 per group); **G** Comparison of the proportion of effector T cells (CD8^+^IFN-γ^+^ T cells) between the LvSUV39H1 group and LvCtrl group (*n* = 3 per group); **H** Flow cytometry analysis of SASP secretion in the LvSUV39H1 group and LvCtrl group (*n* = 3 per group). *P < 0.05, **P < 0.01, ***P < 0.001, ****P < 0.0001, ns = no significance.

To explore whether SUV39H1, a downstream gene of LCP2, drives the formation of an immunosuppressive senescent microenvironment in NKTCL cells, we constructed NKTCL cell lines overexpressing SUV39H1 and their control counterparts (LvSUV39H1 and LvCtrl) for subsequent experiments (Fig. S5A). Transcriptome sequencing and KEGG enrichment analysis revealed that differentially expressed genes in the SUV39H1-overexpressing group were significantly enriched in the cellular senescence regulatory network (Fig. 5A).

Next, we validated that LvSUV39H1 cells exhibited senescence-associated chemoresistance using a series of biological function assays. First, the CCK-8 assay was used to evaluate drug resistance in LvSUV39H1 and LvCtrl cells. The results showed that compared to LvCtrl cells, the inhibition rate of ADM-treated LvSUV39H1 cells was significantly reduced (Fig. S5B). A similar trend was observed following treatment with gemcitabine, cisplatin, oxaliplatin, L-asparaginase, and bendamustine (Fig. S6A–E). Second, SA-β-gal staining was performed to compare cellular senescence, and the results indicated that the senescence rate of LvSUV39H1 cells was significantly higher than that of LvCtrl cells (Fig. S5C). RT-qPCR was used to detect the expression of SASP factors in LvSUV39H1 cells, which revealed that IL-6 and MMP9 were significantly upregulated in both cell lines (Fig. S5D). Flow cytometry was used to assess the apoptotic effects of ADM on LvSUV39H1 and LvCtrl cells. After treatment with 2 μg/mL ADM for 72 h, the apoptosis rate of LvSUV39H1 cells was significantly lower than that of LvCtrl cells (Fig. S5E). Similarly, flow cytometric analysis of the cell cycle showed that a higher proportion of LvSUV39H1 cells were arrested in the G0/G1 phase than LvCtrl cells (Fig. S5F).

It has been reported that SUV39H1 is a key regulator of T cell exhaustion [21, 22]. To investigate the effect of LvSUV39H1 cells on the immunosuppressive microenvironment and SASP secretion, we co-cultured PBMCs from healthy donors with LvSUV39H1 or LvCtrl cells (Fig. 5B). Flow cytometry revealed significant differences between the LvSUV39H1 and LvCtrl group in the proportions of multiple T cell subsets, including CD3^+^CD69^+^, CD3^+^PD-1^+^, CD3^+^LAG3^+^, CD3^+^TIM3^+^ T cells, as well as Treg cells and CD8^+^IFN-γ^+^ T cells (Fig. 5C–G). Similarly, flow cytometry-based SASP detection revealed that the cytokine IL-8 was highly expressed in both SNT16-LvSUV39H1 and NKYS-LvSUV39H1 cells compared to their respective LvCtrl counterparts (Fig. 5H).

### Targeting LCP2-mediated IQGAP2/LaminA/C/SUV39H1 axis exerts a vital effect on the clearance of senescent cells and overcomes chemoresistance in NKTCL

**Fig. 6:**
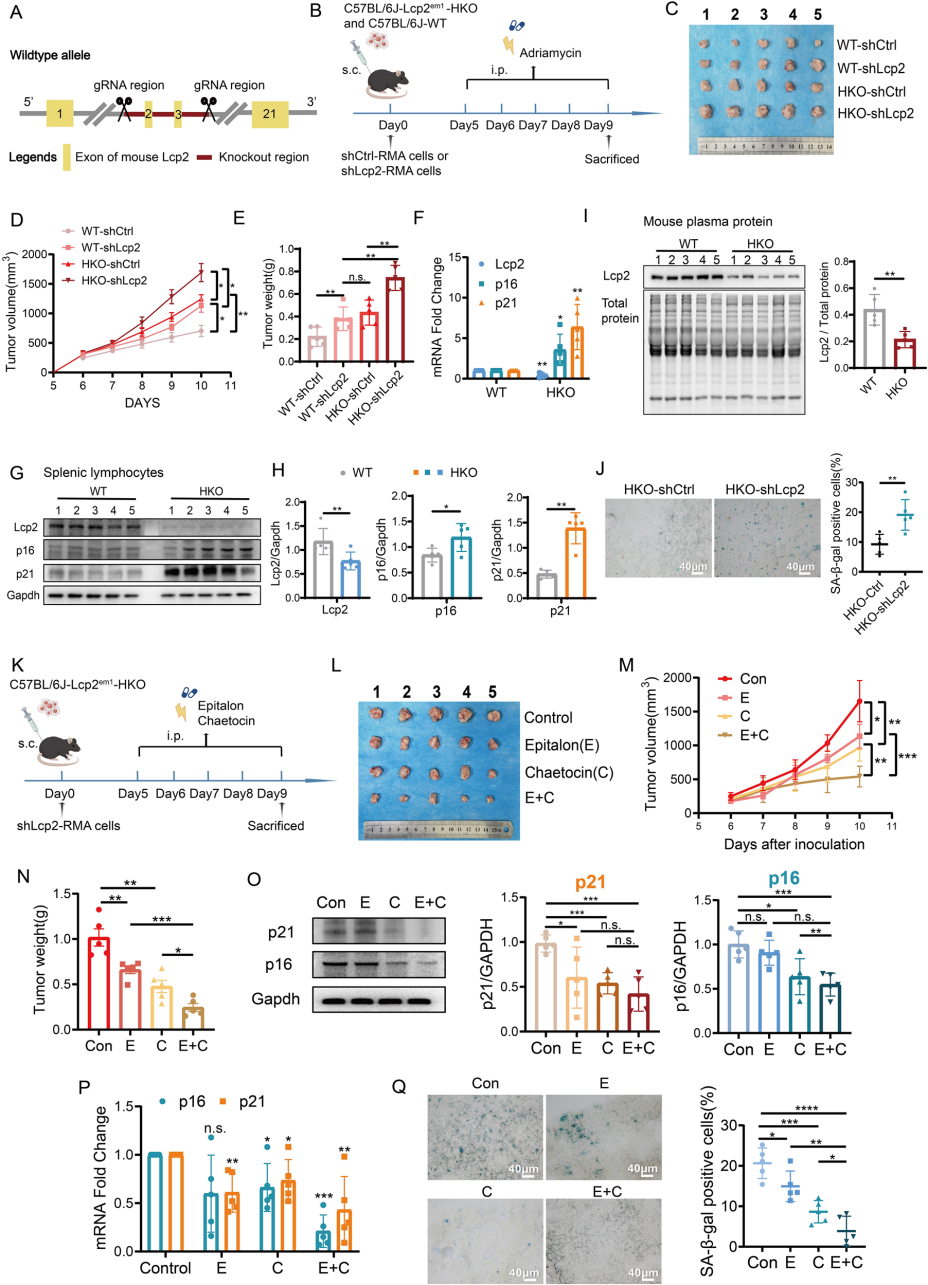
Intervention against the LCP2-mediated IQGAP2/LaminA/C/SUV39H1 axis is essential for facilitating senescent cell clearance and overcoming chemoresistance in NKTCL. **A** Overview of the Lcp2-KO targeting strategy; **B** Schematic diagram illustrating the construction of the NKTCL senescent microenvironment model and detection of its chemoresistance; **C**–**E** Schematic diagrams showing tumour growth and tumour weight in HKO-shLcp2 mice and their control groups (*n* = 5 per group); **F**–**H** RT-qPCR and WB detection of Lcp2/p16/p21 expression in splenic lymphocytes from WT and HKO mice, with bar graph statistical analysis (*n* = 5 per group); **I** WB detection of Lcp2 expression in peripheral plasma from WT and HKO mice, using statistical analysis (*n* = 5 per group); **J** SA-β-gal staining to detect cellular senescence in subcutaneous tumours from HKO-shCtrl and HKO-shLcp2 mice (*n* = 5 per group); **K** Schematic diagram illustrating the reversal of senescence-associated chemoresistance in the NKTCL senescent microenvironment model (HKO-shLcp2 mice); **L**–**N** Therapeutic effects of Epitalon and Chaetocin on tumours in HKO-shLcp2 mice, including tumour growth curves and tumour weight schematics (*n* = 5 per group); **O**, **P** WB and RT-qPCR detection of p16/p21 expression in splenic lymphocytes from HKO-shLcp2 mice after different drug treatments, with bar graph statistical analysis (*n* = 5 per group); **Q** SA-β-gal staining to assess the alleviation of cellular senescence in subcutaneous tumours from HKO-shLcp2 mice after different drug treatments (*n* = 5 per group); *P < 0.05, **P < 0.01, ***P < 0.001, ****P < 0.0001, ns = no significance.

Our previous experiments confirmed the phenomenon and mechanism through which low LCP2 expression induces senescence-associated chemoresistance in NKTCL. To further validate this, we utilised Lcp2 knockout mice (Fig. 6A) and established a subcutaneous tumour model with shLcp2-RMA cells to construct an animal model of a drug-resistant senescent microenvironment. Since homozygous Lcp2 knockout is lethal in mice, we used heterozygous C57BL/6J-Lcp2^em1^-HKO mice (HKO) to simulate a state of low LCP2 expression in patients with drug resistance after systemic chemotherapy. Additionally, we used senolytics to clear senescent cells, remodel the immunosuppressive senescent microenvironment, and reverse drug resistance.

We first evaluated the drug resistance of the animal model using ADM. Mice were divided into four groups: wild-type (WT) mice bearing subcutaneous shCtrl-RMA or shLcp2-RMA tumours (designated as WT-shCtrl and WT-shLcp2, respectively) and HKO mice bearing subcutaneous shCtrl-RMA or shLcp2-RMA tumours (designated as HKO-shCtrl and HKO-shLcp2, respectively). Results showed that HKO-shLcp2 mice exhibited significantly greater drug resistance than the other three groups (Fig. 6B–E). RT-qPCR and WB analyses were used to detect the expression of p21 and p16, which are indicators of cellular senescence in animal models. Compared to the other three groups, HKO-shLcp2 mice showed increased p16 and p21 expression in splenic lymphocytes (Fig. 6F–H). Furthermore, WB analysis revealed that plasma Lcp2 protein expression was significantly lower in HKO mice than in WT mice (Fig. 6I). SA-β-gal expression in subcutaneous tumours was higher in HKO-shLcp2 mice than in HKO-shCtrl mice (Fig. 6J). These results confirmed that HKO-shLcp2 mice exhibited characteristics of NKTCL senescence-associated chemoresistance.

We selected a combination of Epitalon (an inhibitor of the Lcp2 downstream cellular senescence regulatory network) and Chaetocin (an inhibitor of the key senescence-related molecule Suv39h1 downstream of Lcp2) [23–25] to clear senescent NKTCL cells, with the aim of reversing the drug-resistant microenvironment. The results showed that the Epitalon plus Chaetocin (E + C) group had a significantly better therapeutic effect on tumours in HKO-shLcp2 mice than the single-agent and control groups (Fig. 6K–N). RT-qPCR and WB analyses demonstrated that p21 and p16 expression in the splenic lymphocytes of HKO-shLcp2 mice was significantly lower in the E + C group than in the single-agent and control groups (Fig. 6O, P). Additionally, SA-β-gal expression in subcutaneous tumours of mice in the E + C group was lower than in the single-agent and control groups (Fig. 6Q). These findings confirm that the combination of Epitalon and Chaetocin effectively reversed senescence-associated chemoresistance in NKTCL cells.

**Fig. 7:**
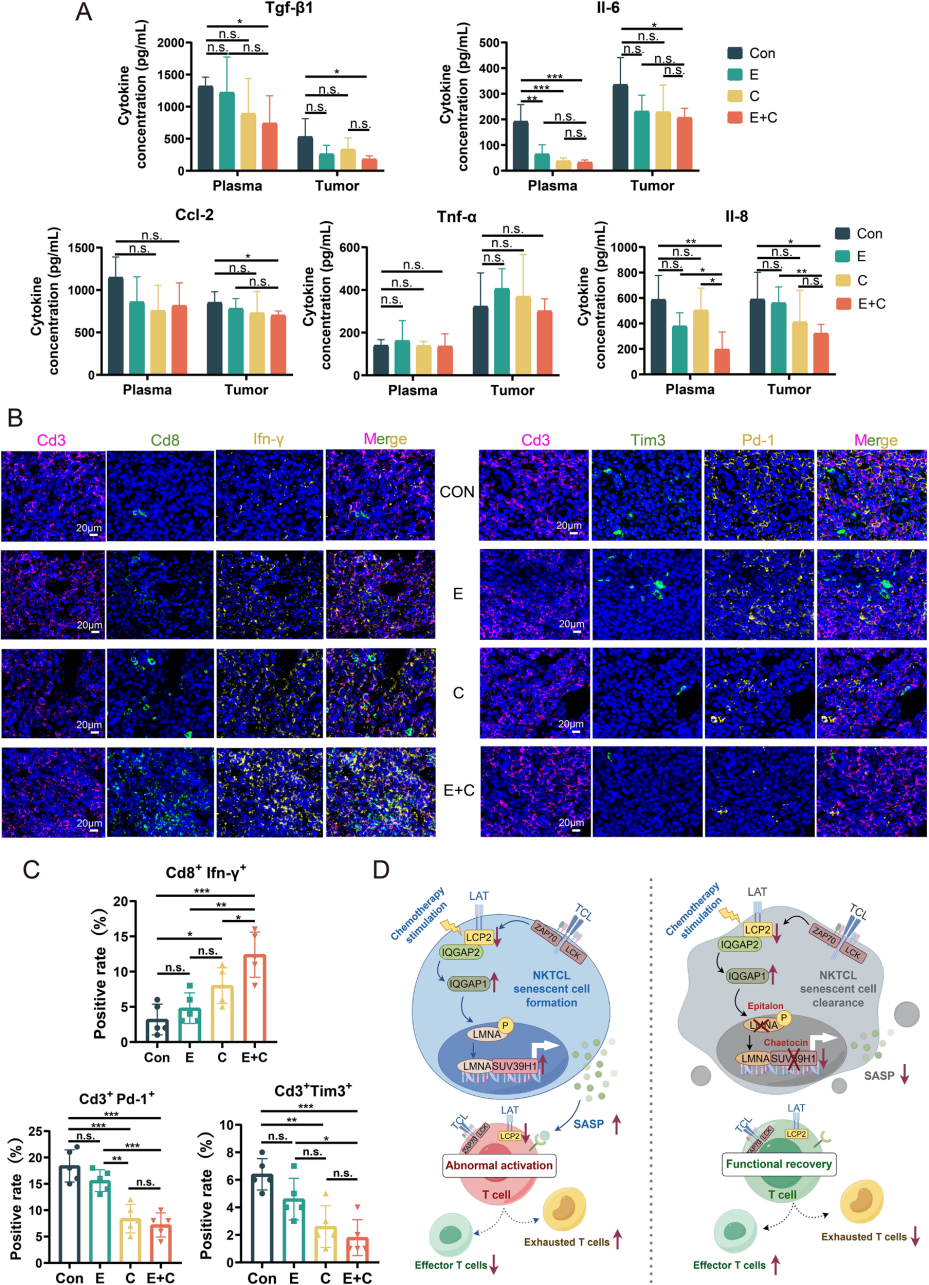
Inhibition of the LCP2-mediated IQGAP2/LaminA/C/SUV39H1 axis is crucial for ameliorating the immunosuppressive state within the ageing microenvironment in vivo. **A** ELISA assay to measure the levels of SASP factors (Tgf-β, Il-6, Ccl-2, Tnf-α, Il-8) in tumour tissues and plasma from a mouse model of HKO-shLcp2 (*n* = 5 per group); **B**, **C** The representative images and bar graphs of Cd8^+^ Ifn-γ^+^ cells, Cd3^+^ Pd-1^+^ cells, and Cd3^+^ Tim3^+^ cells in tumours of HKO-shLcp2 mice, as determined by mIHC (*n* = 5 per group); **D** Schematic diagram illustrating the mechanism by which LCP2 mediates the development of senescence-associated chemoresistance and the role of inhibitors targeting the LCP2 downstream senescence regulatory network (Epitalon) and SUV39H1 (Chaetocin) in reversing the immunosuppressive state induced by senescence-associated chemoresistance in NKTCL. *P < 0.05, **P < 0.01, ***P < 0.001, ns = no significance.

To explore the effect of Epitalon plus Chaetocin on the alleviation of immunosuppression in the NKTCL senescent microenvironment, ELISA and mIHC assays were performed. Relative to both the control and monotherapy groups, the E + C group exhibited significantly reduced expression of key SASP factors (Tgf-β1, Il-6, Ccl-2, Il-8) in tumour tissue and plasma of HKO-shLcp2 mice (Fig. 7A). Tnf-α levels showed no significant difference across groups. Furthermore, the E + C group demonstrated a significantly elevated proportion of Cd8^+^ Ifn-γ^+^ T cells and a concomitant reduction in Cd3^+^Pd-1^+^ and Cd3^+^ Tim3^+^ T cells compared to the control and monotherapy groups (Fig. 7B).

In summary, repeated stimulation with chemotherapeutic drugs downregulates LCP2 expression in NKTCL and normal T cells, activates the IQGAP2/LaminA/C/SUV39H1 axis, releases SASP, promotes the expression of Tregs and exhausted T cells, and inhibits effector T cell function, thereby forming an immunosuppressive microenvironment associated with drug resistance. Inhibitors targeting the LCP2 downstream senescence regulatory network (Epitalon) and SUV39H1 (Chaetocin) effectively reversed the immunosuppressive state of senescence-associated chemoresistance (Fig. 7C).

## Discussion

TIS, a state of irreversible cell cycle arrest induced by chemotherapy, has emerged as a key driver of chemoresistance and tumour recurrence across malignancies. In lymphoma, TIS promotes the secretion of SASP factors, fostering an immunosuppressive microenvironment and enhancing stemness traits [20, 26, 27]. Recent studies in diffuse large B-cell lymphoma showed that ADM-induced TIS upregulates stemness genes and activates NF-κB-mediated SASP, promoting resistance to subsequent therapies [9, 28]. In NKTCL, our study reveals chemo-resistant NKTCL cells have distinct TIS features, including elevated SA-β-gal activity, SUV39H1 expression, and SASP factors.

TCR adaptor protein LCP2, also known as SLP76, is ectopically expressed in chronic lymphocytic leukaemia cells, with expression levels varying among patients [29]. SLP76 deficiency has been associated with an increased susceptibility to severe EBV infection; notably a child with a novel SLP76 mutation succumbed to EBV-related lymphoma, suggesting a potential role of SLP76 in lymphoma development [30]. SLP76 mediates T cell senescence by integrating signals from antigen and cytokine receptors to regulate cellular senescence programmes [31]. These studies suggest an important role of LCP2 in mediating lymphomagenesis and T cell senescence. However, the specific role of LCP2 in drug resistance in NKTCL has not been previously reported. Our research initially found that LCP2 is downregulated in the plasma of patients with R/R NKTCL and NKTCL drug-resistant cells, which correlates inversely with senescence marker SA-β-gal. Mechanistically, LCP2 loss activates the regulatory network of DNA damage-induced senescence.

The histone H3K9 methyltransferase, SUV39H1, drives heterochromatin formation, stem cell-like characteristics and senescence in lymphoma [9]. In melanoma, SUV39H1 promotes T-cell exhaustion by silencing IFN-γ responsive genes [21]. Recent studies have suggested that SUV39H1 and LaminA/C intersect functionally to maintain genome stability and heterochromatin organisation. This interplay is critical for preserving genomic stability and regulating cellular senescence [32]. Our study extends these findings to NKTCL. Phosphoproteomics data show that the LaminA/C and SUV39H1 axes, regulated by LCP2, constitute a novel molecular network that drives cellular senescence. SUV39H1 bridges senescence and immune evasion in NKTCL, promoting chemotherapy resistance in the ageing microenvironment and underscoring NKTCL’s unique reliance on this senescence marker.

Currently, in the study of TIS, animal models employed in existing research include the transgenic p16-3MR mouse model expressing a senescence reporter system used by Maggiorani D et al. [33], as well as xenograft mouse models constructed using shRNA-modified tumour stem cells exhibiting a senescent phenotype, as reported by Wang MJ et al. [34]. Our model integrates the Lcp2 gene knockout mouse heterozygote model (HKO mice)-which is consistent with the heterozygous animal models reported in previous studies [35]-with shLcp2-RMA cells to establish a subcutaneous xenograft tumour model, characterised by decreased Lcp2 protein expression, an increased SA-β-gal positivity rate, and enhanced chemoresistance. This model surpasses traditional xenografts by integrating the host genetic background (Lcp2 status) with tumour cell senescence, simulating the low expression state of LCP2 in the tumours and plasma of patients after repeated chemotherapy and ageing.

In recent years, there has been growing interest in developing senolytic drugs that specifically target and eliminate senescent cells, with the aim of disrupting the pro-tumorigenic functions of the ageing microenvironment [36, 37]. Among them, it is reported that SUV39H1 inhibitor Chaetocin restores the therapeutic sensitivity and reverses resistance to anticancer treatments [25, 38]. Epitalon has been demonstrated to exhibit tumour-suppressive [39] and anti-cellular senescence [40] activities. Our study focused on evaluating the combination of Epitalon and Chaetocin as potential senolytic agents in chemotherapy-resistant NKTCL patients, which promotes an anti-tumour immune response. The translational specificity of this strategy is reflected in two main aspects. First, tumour-specific relevance of the targets: In tumour cells from patients with R/R NKTCL, activation of the IQGAP2/LaminA/C/SUV39H1 axis triggered by low LCP2 expression represents a specific molecular event. Therefore, inhibitors targeting this pathway (Epitalon and Chaetocin) can selectively eliminate senescent cells in R/R NKTCL, thereby partially overcoming chemotherapy resistance. Second, advantages for clinical translation of the drug combination: Both Epitalon and Chaetocin have been previously reported as anti-senescence and anti-tumour agents. This study confirms that their synergistic combination significantly enhances therapeutic efficacy in R/R NKTCL, with a well-defined mechanism of action targeting functional nodes downstream of LCP2, providing a solid experimental basis for subsequent for subsequent clinical translation strategies.

Additionally, supplementing the LCP2 protein to suppress the activation of the cellular senescence network, thereby partially eliminating therapy-induced senescent cells and potentially enhance response to chemotherapeutics, may represent a promising therapeutic strategy. However, as previously reported, the development of LCP2 has been impeded by its “undruggable” chemical structure [18], which poses a significant challenge that must be overcome for the future development of commercialised drugs.

It should be noted that in this study, ADM was employed solely as a canonical TIS inducer [13, 14] and a multidrug resistance inducer [9, 12]. Its primary purpose was to explore the mechanisms of senescence-associated resistance in both in vitro and in vivo models, rather than to provide a theoretical basis for reintroducing anthracyclines into the clinical treatment of NKTCL. Currently, the clinical management of NKTCL is well-established, primarily relying on L-asparaginase-containing regimens, gemcitabine, and platinum-based agents [6]. Given the intrinsic resistance of NKTCL to anthracyclines, these drugs are now rarely used in its clinical therapy [6]. Therefore, in this work, ADM served merely as a tool compound, providing a robust experimental foundation for investigating the mechanisms of TIS-associated multidrug resistance.

Our study still has some limitations. First, SASP factor expression has heterogeneity [41], which varies among different NKTCL cell lines and T cells (Fig. 1D). Second, the mechanism of LCP2 downregulation in the plasma remains unclear. The potential pathways may include proteolytic cleavage or exosomal secretion. Third, our study preliminarily identified co-expression of the cytoplasmic molecule IQGAP2 and the nuclear molecule LaminA/C downstream of LCP2; however, the precise phosphorylation-related regulatory mechanisms warrant further investigation. Finally, future studies need to prospectively collect more paired tumour tissue and liquid biopsy samples from patients before treatment, at remission, and at recurrence in large-scale multicenter clinical trials, and integrate transcriptomic, proteomic, and epigenomic analyses to more deeply elaborate and verify the dynamic evolution process of TIS in the drug resistance of NK/T cell lymphoma and its potential for targeted therapy.

In conclusion, the core contribution of this study lies in defining LCP2 as a pivotal regulator of TIS-related chemoresistance in NKTCL. It unveils the IQGAP2/LaminA/C/SUV39H1 axis as a potential therapeutic target, thereby providing a novel focus for subsequent research into TIS-associated resistance mechanisms and clinical monitoring. This work demonstrates that targeting this pathway with senolytics (Epitalon) and SUV39H1 inhibitors (Chaetocin) holds promise for overcoming resistance—particularly the senescence-related multidrug resistance induced by contemporary first-line chemotherapy regimens. These findings offer a new strategic direction and experimental foundation for addressing the clinical challenge of chemoresistance in NKTCL, warranting further translational investigation.

## Supplementary information

Supporting Information 1

Supporting Information 2

Supporting Information 3

Original Data File of Western Blotting

## Data Availability

The mass spectrometry proteomics data have been deposited to the ProteomeXchange Consortium (https://proteomecentral.proteomexchange.org) via the iProX partner repository [42] with the dataset identifier PXD073967, PXD073969 and PXD073971. The raw mRNA sequencing data have been deposited in the NCBI BioProject database under the accession number PRJNA1417292.
