# Supplementary material for: LCP2 mediates SUV39H1-driven cellular senescence-related chemoresistance in natural killer/T-cell lymphoma
Source: Cell Death Dis. 2026 May 28;17(1):662. doi: 10.1038/s41419-026-08897-6 (PMC13408427; doi:10.1038/s41419-026-08897-6)
Supplement: Supplementary file 1 — Supporting Information 1 [file 41419_2026_8897_MOESM1_ESM.docx]

**Supplementary materials and methods**

1. **Cell lines and culture**

The KHYG-1, NKYS, SNT-16, and RMA cell lines were cultured in RPMI-1640 medium (Gibco, USA) containing 10% fetal bovine serum (FBS, Gibco, USA) and 1% penicillin/streptomycin (Gibco, USA). NKYS and KHYG-1 were supplemented with 100 IU/mL recombinant human interleukin-2-dependent (rhIL-2; SLPHARM, China). SNK-6 cells were cultured in X-VIVO medium (Lonza, Basel, Switzerland) supplemented with 10% FBS, 1% penicillin/streptomycin, and 600 IU/mL rhIL-2. NKYS and KHYG-1 cell lines were kindly provided by Dr. Wing C. Chan (City of Hope Medical Center). SNK-6 cell line was kindly provided by Dr. Norio Shimizu of Chiba University. The SNT16 cell line was obtained from Guangzhou Bairui Biomedical Technology Co., Ltd. (Guangzhou, China). The RMA cell line was purchased from Shanghai WheLab Biotechnology Co., Ltd. (Shanghai, China).

Resistance to adriamycin (ADM, Selleck, USA) was induced in the KHYG-1, NKYS, SNK-6, and SNT-16 cell lines by gradually increasing the ADM concentration in the culture medium, starting with an initial concentration of 50 ng/mL. The resistance index (RI) was calculated using the following formula: RI = IC50_(NKTCL/ADM)_/IC50_(NKTCL)_[1].

1. **Senescence-associated β-galactosidase (SA-β-gal) staining**

Cells were collected by centrifugation into 1.5 mL centrifuge tubes, or frozen sections were attached to adherent slides and then washed with PBS. 1 mL β-galactosidase staining fixative solution was added into centrifuge tubes and fixed at room temperature (RT) for 15 min with shaking slowly on a shaker during fixation. Cells or tissue sections were washed three times with PBS. 0.5-1 mL staining solution was added into per tube (staining solution A: staining solution B: staining solution C: X-Gal solution = 10 μL: 10 μL: 930 μL: 50 μL)(Beyotime, China). The cells or tissue sections were incubated overnight at 37 °C without CO_2_. Stained cells were fixed onto slides. Images were captured using a microscope (Olympus CX23, Japan).

**3. Detection of the inhibition rate of chemotherapeutic drugs**

Cells were seeded at a density of 1×10⁴ cells/well in 96-well plates and cultured in 90 µL of RPMI-1640 medium (supplemented as described above) at 37 °C.

Subsequently, cells were treated with 10 µL of ADM per well at a concentration gradient for 48 h; cells were treated with 10 µL of gemcitabine, cisplatin, oxaliplatin, L-asparaginase, and bendamustine (all from MCE, USA) per well at a concentration gradient for 72 h.

Cell viability was assessed using a Cell Counting Kit-8 (CCK-8) (UElandy, China) according to the manufacturer's protocol. Absorbance was measured at 450 nm using a Multiskan FC microplate reader (Thermo Fisher Scientific). The inhibition rate was calculated as follows: the inhibition rate (%) = [(OD_control_ - OD_treatment_)/OD_control_] × 100. The resistance index (RI) was calculated using the following formulas: RI = IC50_(NKTCL-shLCP2)_/IC50_(NKTCL-shCtrl)_ and RI = IC50_(NKTCL-LvSUV39H1)_/IC50_(NKTCL-LvCtrl)_. The experiments were independently repeated at least three times.

1. **Reverse transcription quantitative polymerase chain reaction (RT-qPCR)**

Total RNA was extracted using the TRIzol reagent (CWBIO, China) following the manufacturer’s protocol. RNA concentration and purity were assessed using a NanoDrop spectrophotometer (Thermo Fisher Scientific, USA). cDNA synthesis was performed with 1 μg RNA using RT mix with DNase (US EVERBRIGHT, China). PCR was performed on a CFX384 Real-Time System (BIO-RAD, USA) using Universal SYBR Green qPCR Supermix (US EVERBRIGHT, China) following the manufacturer’s protocol. Relative expression was calculated using the 2^(-ΔΔCt) method with GAPDH. The primer information is detailed in the Supporting Information 2 (Table S1).

1. **Western blotting (WB)**

Total protein was extracted using RIPA buffer (CWBIO, China) supplemented with protease and phosphatase inhibitors (Beyotime, China). Plasma proteins were extracted using a plasma protein extraction kit (Solarbio, China), and the interference from albumin was removed using Minute™ Albumin Depletion Reagent (Invent, USA), following the manufacturers' protocols.

The protein concentration was determined using a BCA assay (CWBIO, China). Equal amounts of protein were separated via SDS-PAGE (EpiZyme, China) or the TGX Stain-Free FastCast Acrylamide Kit (BIO-RAD, USA) and transferred to PVDF membranes (BIO-RAD, USA). Membranes were blocked with 5% bovine serum albumin in tris-buffered saline with Tween 20 for 2 h, incubated with primary antibodies overnight at 4 °C, washed, and probed with HRP-conjugated secondary antibodies for 1 h at RT. The bands were visualised using an ECL substrate (CWBIO, China) and captured using a ChemiDoc XRC system (BIO-RAD, USA). The antibody information is listed in the Supporting Information 2 (Table S2).

1. **TMT-labelled quantitative proteomics**

Total proteins from KHYG1-ADM and KHYG1 cells were extracted using RIPA buffer supplemented with protease inhibitors. Protein concentration was determined via BCA assay. Equal amounts of protein from each sample were reduced with 5 mM dithiothreitol at 56 °C for 1 h, alkylated with 10 mM iodoacetamide in the dark for 45 min, and precipitated with cold acetone overnight.

Tryptic peptides were initially dissolved in 0.5 M triethylammonium bicarbonate. The peptides in each channel were labelled with their corresponding TMT reagents according to the manufacturer’s protocol (Thermo Fisher Scientific, USA) and incubated at RT for 2 h. A 5 μL aliquot from each sample was combined, desalted, and subjected to mass spectrometry analysis to assess the labelling efficiency. After confirming labelling efficiency, the samples were quenched by the addition of 5% hydroxylamine. The pooled samples were desalted using a Strata X C18 solid-phase extraction column (Phenomenex, USA) and dried via vacuum centrifugation.

Liquid Chromatography-Tandem Mass Spectrometry (LC-MS/MS) analysis. Peptides were dissolved in LC mobile phase A and separated using a Vanquish Neo ultra-high-performance liquid chromatography system. Mobile phase A consisted of an aqueous solution containing 0.1% formic acid and 2% acetonitrile, whereas mobile phase B was composed of 0.1% formic acid and 90% acetonitrile in water. Separated peptides were injected into the NSI ion source for ionisation and then analysed using an Orbitrap Exploris™ 480 mass spectrometer (Thermo Fisher Scientific, USA).

1. **Blood-data-independent acquisition (DIA) quantitative proteomics**

Plasma samples were collected from the same patients at The First Affiliated Hospital of Zhengzhou University between June 1, 2022 and March 1, 2023, covering the period from complete remission (CR) to relapsed/refractory (R/R) disease.

Patients diagnosed with natural killer/T-cell lymphoma (NKTCL) enrolled in this study had received L-asparaginase‑containing treatment regimens.

The cellular debris from 10 plasma samples was removed by centrifugation at 12,000 g at 4℃ for 10 min. The supernatant was then transferred to a new centrifuge tube. The top 14 high-abundance proteins were depleted using the Pierce™ Top 14 Abundant Protein Depletion Spin Columns Kit (Thermo Fisher Scientific, USA). Finally, the protein concentration was measured using a BCA kit following the manufacturer’s instructions.

The protein solution was reduced with 5 mM dithiothreitol at 56 °C for 30 min and then alkylated with 11 mM iodoacetamide at RT for 15 minutes in the dark. The protein sample was subsequently diluted by adding 100 mM triethylammonium bicarbonate to adjust the urea concentration to less than 2 M. Trypsin was then added at a trypsin-to-protein mass ratio of 1:50 for an initial overnight digestion, followed by a second digestion with trypsin at a ratio of 1:100 for 4 h. The resulting peptides were desalted using a C18 solid-phase extraction column.

Tryptic peptides were dissolved in solvent A (0.1% formic acid, 2% acetonitrile in water) and directly loaded onto a home-made reversed-phase analytical column (25 cm length, 75/100 μm i.d.). Peptide separation was performed using a NanoElute UHPLC system (Bruker Daltonics, Germany) at a constant flow rate of 450 nL/min with a gradient of solvent B (0.1% formic acid in acetonitrile): 6%-24% over 70 min, 24%-35% over 14 min, an increase to 80% over 3 min, and a final hold at 80% for 3 min.

Peptides were introduced into a TimsTOF Pro mass spectrometer (Bruker Daltonics, Germany) using a capillary source. An electrospray voltage of 1.60 kV was used. Both the precursor and fragment ions were detected using a TOF detector with an MS/MS scan range of 100-1700 m/z. The timsTOF Pro was operated in the parallel accumulation serial fragmentation mode. Precursor ions with charge states ranging from 0-5 were selected for fragmentation and 10 PASEF-MS/MS scans were acquired per cycle. The dynamic exclusion was set at 30 s.

1. **4D-Fast DIA quantifies phosphorylation-modified proteomes**

Protein Extraction: Samples were cryogenically ground in liquid nitrogen and lysed with four volumes of buffer containing 1% SDS, 1% protease inhibitors, and 1% phosphatase inhibitors. Lysates were sonicated on ice for 3 min, clarified by centrifugation (12,000 g, 4 °C, 10 min), and protein concentration was determined via BCA assay.

Trypsin Digestion: Proteins were precipitated with 20% trichloroacetic acid at 4 °C for 2 h, pelleted (4,500 g, 4 °C, 5 min), washed thrice with cold acetone, and resuspended in 200 mM triethylammonium bicarbonate. Trypsin digestion (1:50 enzyme:protein) was performed overnight, followed by reduction (5 mM dithiothreitol, 56 °C, 30 min) and alkylation (11 mM iodoacetamide, RT, 15 min in the dark). The peptides were then desalted using a Strata X SPE column.

Affinity Enrichment: Phosphopeptides were enriched using immobilised metal-affinity chromatography microspheres. The samples were incubated with IMAC beads in loading buffer (50% acetonitrile/0.5% acetic acid), washed sequentially with 50% acetonitrile/0.5% acetic acid and 30% acetonitrile/0.1% trifluoroacetic acid, and eluted with 10% NH_4_OH.

Mass Spectrometry: Peptides were separated on a 25 cm, 100μm i.d. reverse-phase column using a gradient of 3-80% solvent B (0.1% formic acid, 90% acetonitrile) over 30 min at 700 nL/min (EASY-nLC 1200). The Orbitrap Exploris 480 settings included an electrospray voltage of 2300 V, FAIMS CV of 45 V, MS1 resolution of 120,000 (350-1400 m/z), MS/MS resolution of 45,000 (fixed first mass of 120 m/z), HCD collision energy of 27%, and AGC target 1×10⁶.

Database Search: Data-dependent acquisition spectra were processed using SpectronAut v17.0/Pulsar against Homo sapiens_9606_SP_20230103. fasta (20,389 entries and decoy databases). Parameters included a max 2 missed cleavages, fixed carbamidomethylation of Cys, variable modifications (N-terminal acetylation, Met oxidation, Ser/Thr phosphorylation), and FDR <1% for proteins, peptides, and PSMs. The generated spectral library was used for DIA analysis with retention time prediction using nonlinear correction.

1. **Construction of NKTCL stable cell lines**

Short hairpin RNAs (shRNAs) were used to construct lentiviral plasmids for the LCP2 and SUV39H1 knockdown. For SUV39H1 overexpression, a recombinant plasmid containing the full-length cDNA was synthesised and cloned into a lentiviral vector (GeneChem, Shanghai, China). Lentiviral particles were packaged into HEK293T cells and used to transduce NKTCL cell lines (NKYS, SNT16, and RMA). The transduced cells were selected using puromycin (Biosharp, China) and blasticidin (MACKLIN, China) to establish stable cell lines. Knockdown and overexpression efficiencies were validated using WB.

All plasmids used in this study were purchased from Shanghai GeneChem Co., Ltd., and the detailed information is as follows:

1. Human LCP2 knockdown plasmids (shRNA) and their control plasmids: The element sequence was U6-MCS-Ubiquitin-Cherry-IRES-puromycin; LCP2-RNAi-1 (sh1-LCP2) Target Seq: CTCAGAAAGTAACAGCAGTTT; LCP2-RNAi-2 (sh2-LCP2) Target Seq: GATCGTTCATTAGCTCCGTTT; LCP2-RNAi-3 (sh3-LCP2) Target Seq: GTGCCGATTCTCAGTAAGTTA; Control sequence: TTCTCCGAACGTGTCACGT;
2. Murine Lcp2 knockdown plasmid (shRNA) and its control plasmid: The element sequence was U6-MCS-Ubiquitin-Cherry-IRES-puromycin; Lcp2-RNAi (sh-Lcp2) Target Seq: CAGTAAGTTGAGTCAAGATAT; Control sequence: TTCTCCGAACGTGTCACGT;
3. Human LCP2 overexpression plasmid and its control plasmid: The element sequence was Ubi-MCS-3FLAG-SV40-Cherry-IRES-Blasticidin;
4. Human SUV39H1 knockdown plasmid (shRNA) and its control plasmid: The element sequence was U6-MCS-Ubiquitin-Cherry-IRES-Blasticidin; SUV39H1-RNAi (shSUV39H1) Target Seq: CCTCGGTATCTCTAAGAGGAA;

(5) Human SUV39H1 overexpression plasmid and its control plasmid: The element sequence was Ubi-MCS-3FLAG-CBh-gcGFP-IRES-puromycin.

**10. RNA sequencing**

Total RNA was extracted using the TRIzol reagent (CWBIO, China) following the manufacturer’s protocol. Genomic DNA was removed using DNase I (Thermo Fisher Scientific). Library Preparation: mRNA was enriched from 1 μg total RNA using poly (A) selection with oligo (dT) magnetic beads. Fragmentation was performed at 94 °C for 15 min. First-strand cDNA was synthesised using random hexamers and reverse transcriptase, followed by second-strand synthesis. Adaptor ligation and PCR amplification were performed. Single-stranded circular DNA molecules generate DNA nanoballs (DNBs), each containing multiple copies of a target sequence. Subsequently, high-quality DNBs were loaded onto the patterned nanoarrays using high-intensity DNA nanochip technology and subjected to sequencing via combinatorial probe anchor synthesis. Subsequent bioinformatics analyses were performed using the <https://biosys.bgi.com/.>

**11.Cell cycle and apoptosis assays**

Cell cycle: The cells were harvested and centrifuged at 250 g for 5 min. The supernatant was discarded, and cells were fixed with 70% cold ethanol and incubated at 4 °C overnight. After fixation, cells were resuspended in PBS containing propidium iodide (PI) (US EVERBRIGHT, China)**,** then incubated at 35 °C for 30 min in the dark to stain DNA. Samples were analysed using flow cytometry.

Cells were resuspended in 1× binding buffer at a density of 1×10^6^ cells/mL. Then, 100 μL of the cell suspension was mixed with 5 μL of Annexin V and 5 μL of 7-AAD (US EVERBRIGHT, China) in the dark for 25 min at RT. The mixture was then washed twice with 1×binding buffer to remove excess conjugates. Samples were analysed using flow cytometry.

**12.Silver stain, Mass spectrometry and Co-immunoprecipitation (Co-IP) assay**

Silver staining assay: The SDS-PAGE gel was fixed in a solution containing 50% methanol and 10% acetic acid (prepared in deionized water) on a shaker at room temperature for 40 min. After discarding the fixation solution, the gel was washed with 100 mL of 30% ethanol for 10 min with shaking (60-70 rpm). The gel was then sensitized by incubation in 100 mL of 1x silver sensitization solution for 2 min with shaking. Following sensitization, the gel was rinsed twice with deionized water for 1 min each. Subsequently, the gel was stained with 100 mL of 1x silver nitrate solution for 10 min with shaking. After staining, the gel was quickly rinsed with 100 mL of deionized water for 1-1.5 min. Development was initiated by adding 100 mL of 1x color development solution (supplemented with 1x development accelerator and incubating for 3-10 min with shaking until desired band intensity was achieved. The reaction was terminated by incubating the gel in 100 mL of 1x stop solution for 10 min. Finally, the gel was rinsed with 100 mL of deionized water for 2-5 min. The stained gel was placed on a white imaging tray and documented.

Mass spectrometry：Protein bands of interest were excised and destained with 30 mM potassium ferricyanide/100 mM sodium thiosulfate. Samples were reduced with 10 mM DTT (56 °C, 45 min) and alkylated with 55 mM iodoacetamide (RT, 30 min in the dark). Samples were digested with trypsin overnight at 37 °C. Peptides were extracted with 50% acetonitrile/5% formic acid and analysed using LC-MS/MS. The information is detailed in the Supporting Information 3.

Co-IP Assay: Cells were lysed in RIPA buffer (Beyotime, China) containing protease/phosphatase inhibitors (Beyotime, China). Cell lysates were incubated with 5-10 μg of primary antibody under gentle agitation at 4 °C overnight. The lysates were added with protein A+G agarose (Beyotime, China) and incubated at 4 °C for 2 h. The beads were washed with RIPA buffer, resuspended in 1×SDS-PAGE loading buffer (CWBIO, China), and heated at 100 °C for 10 min to denature the proteins. The supernatants were collected and subjected to western blot analysis .

**13.Immunohistochemistry (IHC), immunofluorescence (IF) and multiple immunohistochemistry (mIHC)**

IHC: Tissues were trimmed and fixed in formalin for ≥ 3 days, with necrotic tissue removed from the target plane; then placed in embedding cassettes for dehydration, paraffin infiltration and embedding. Paraffin blocks were rough-sectioned then fine-sectioned into 4 μm slices, which were flattened in a thermostatted water bath, mounted on slides and baked at 65 °C for 30 min to prevent detachment. Slides were sequentially deparaffinized and rehydrated in xylene Ⅰ/Ⅱ (20 min each), 100% ethanol, 75% ethanol and distilled water (5 min each). For antigen retrieval, slides were soaked in 0.01 M sodium citrate buffer, microwaved at medium power for 8 min, paused for 7 min, then heated at medium-low power for 7 min, and cooled to room temperature for ≥ 2 h, followed by 3×PBST washes (5 min each) on a shaker. Tissues were circled with an IHC hydrophobic pen and blocked with 3% BSA for 50 min. Proportionally diluted primary antibody was added for overnight incubation at 4 °C in a dark humidified chamber; after 3×PBST washes, secondary antibody was added and incubated at room temperature for 50 min. Slides were washed 3×with PBS, stained with DAB (microscopically monitored), counterstained with hematoxylin for 1 min, rinsed, differentiated in 1% acid alcohol for 2-5 s, blued in 1% ammonia water for 10 s and rinsed. Finally, slides were dehydrated in 100% ethanol for 20 min, cleared in xylene for 5 min, and mounted with neutral balsam for observation. Sections were imaged under a pathological slide imager (3DHISTECH，Hungary).

IF and mIHC: Slides were sequentially immersed in Xylene Ⅰ (20 min), Xylene Ⅱ (20 min), 100% ethanol (5 min), 75% ethanol (5 min) and distilled water (5 min). Slides were soaked in 0.01 M sodium citrate buffer, microwaved at medium power for 8 min, paused for 7 min, then heated at medium-low power for 7 min, and cooled to room temperature for over 2 h. They were washed three times with PBST on a shaker (5 min each), tissues were circled with an IHC hydrophobic pen, and non-specific binding was blocked with 3% BSA for 50 min. Proportionally diluted primary antibody was added, and slides were incubated overnight at 4 °C in a dark humidified chamber, then washed three times with PBST (5 min each) on a shaker. Species-specific secondary antibodies conjugated with different fluorophores were added for incubation at room temperature for 50 min, followed by another three PBST washes (5 min each) on a shaker. Proportionally diluted DAPI was added for staining at room temperature in the dark for 7 min, and slides were washed three times with PBS (5 min each) on a shaker. Mounting and digital scanning: Slides were scanned with a pathological slide imager, and the staining signals were analyzed using CaseView v2.4 software.

Staining reactions were assessed using the immunoreactive score (IRS), calculated as the product of staining intensity (SI) and percentage of positive cells (PP). Scoring criteria were defined as follows: SI grading: 0 (negative), 1 (weak), 2 (moderate), and 3 (strong); PP grading: 0 (negative), 1 (1-10%), 2 (11-50%), 3 (51-80%), and 4 (>80%). Five non-overlapping high-power fields per tumour section were also evaluated. The IRS for each field was calculated by multiplying the SI and PP scores. Specimens were stratified into two groups based on cumulative IRS: low expression (0-2 points) and high expression (≥3 points) [2].

**14.Isolation of peripheral blood lymphocytes (PBMCs) and normal NK/T cells**

Peripheral blood was collected in EDTA-containing tubes and layered over (Tbdscience, China) and centrifuged at 700 g for 30 min at 20 °C. The PBMC layer was aspirated, washed twice with PBS, and resuspended in RPMI 1640 medium.

For NK/T cell isolation, PBMCs were incubated with magnetic beads conjugated to NK/T cell-specific antibodies (CD3/CD56; Miltenyi Biotec, Germany) at 4 °C. The labelled cells were separated using magnetic cell separation.

**15.Co-culture of PBMCs and NKTCL Cell Lines**

PBMCs derived from healthy donors were co-cultured with the SNT16 or NKYS cell lines at a 5:1 ratio. After cultivation for 48 h, the cells were harvested and stained for surface markers to detect immune receptor expression using flow cytometry (antibody information is listed in Supporting Information: Table S2). The supernatant was harvested and stained to detect differences in the expression of secreted cytokines by flow cytometry (BD Biosciences, USA).

The specific procedures were as follows: 50 μL of vortex-mixed capture microsphere suspension and 50 μL of the sample to be tested were added into the reaction tube, mixed thoroughly at room temperature, and incubated in the dark for 1 h; after centrifugation, magnetic separation was performed, 100 μL of fluorescently labeled antibody was added, mixed thoroughly at room temperature, and incubated in the dark for 1 h; after centrifugation, magnetic separation was performed again, 200 μL of bead dilution was added for one wash, magnetic separation was conducted once more, 200-300 μL of bead dilution was added to resuspend the microspheres, and detection was performed by flow cytometry [3].

**16.Haematoxylin & Eosin (HE) Staining**

De-paraffinised tissue sections were rehydrated using a graded ethanol series (100%, 95%, 80%, and 70%) for 5 min each, and then rinsed with distilled water. Sections were stained with a haematoxylin solution for 5-8 min, followed by differentiation in 1% hydrochloric acid-ethanol for 30 s to remove excess stain.

After rinsing with tap water to blue the nuclei, the sections were counterstained with an eosin solution for 2-3 min. The samples were dehydrated using a reverse ethanol series and cleared in xylene for 5 min. Finally, the sections were mounted with neutral balsam and observed under a light microscope (Olympus CX23, Japan).

**17.Enzyme-Linked Immunosorbent Assay (ELISA)**

Recombinant standard proteins, the tumour homogenate supernatant (1 mL PBS: 100 mg tissue) and plasma were added to pre-coated Il-6/Tgf-β/Tnf-α/Il-8/Ccl-2 ELISA plates (MULTI SCIENCES/Elabscience, China) and incubated following the manufacturers' protocols. Biotinylated antibodies were then added to each well. A streptavidin-horseradish peroxidase (HRP) conjugate was added and incubated, followed by five washes to remove excess conjugate. Finally, TMB substrate was added, and the plates were incubated in the dark. The reaction was terminated using a stop solution. The absorbance at 450 nm was measured using a microplate reader (Thermo Fisher Scientific, USA). Concentrations were calculated based on a standard curve.

**18.Tumour‑bearing mouse model**

6-8 week-old C57BL/6J mice (GemPharmatech, China) were used in this study. Animals were randomly assigned to groups, with at least five animals per group. RMA cells were resuspended at 1×10⁷ cells/mL in PBS, and 200 μL of cell suspension (1×10⁶ cells) was injected subcutaneously into the right flank of each mouse. After tumour formation, the mice were intraperitoneally injected (i.p.) with ADM (5 mg/kg, once daily; Selleck, USA).

**19.Establishment of the mouse model of drug-resistant ageing microenvironment**

A CRISPR/Cas9-based approach was employed to generate Lcp2 knockout mice on a C57BL/6J background (C57BL/6J-Lcp2^em1^) (Cyage, China). The Lcp2 gene (NM_010696; ENSMUSG00000002699) on chromosome 11 contains 21 exons. Exons 2-3, encoding a 110-bp critical domain, were targeted. Ribonucleoproteins complexed with Cas9 and guide RNAs were microinjected into the fertilised eggs. Founder mice were genotyped using PCR and Sanger sequencing to confirm the presence of frameshift mutations. Cross-positive F0 mice and wild-type C57BL/6 mice were used to obtain heterozygous F1 offspring. Intercross F1 to generate F2 mice.

Tail biopsies (2-3 mm) were collected from 2-3-week-old mice under anaesthesia and immediately immersed in a lysis buffer containing proteinase K. DNA was extracted using a Mouse Tail Genotype Identification kit (Beyotime, China). Genotyping PCR was performed using a QX200 PCR System (BIO-RAD, USA). Wild-type and heterozygous mice were distinguished based on their band sizes on agarose gels. To validate the Lcp2 knockdown, protein expression was detected via WB, and functional phenotypes were assessed in mice.

6-8 week-old F3 C57BL/6J-Lcp2^em1^ mice were used in this study and randomized into experimental groups with at least five animals per group. RMA cells were resuspended at 1×10⁷ cells/mL in PBS, and 200 μL of cell suspension (1×10⁶ cells) was injected subcutaneously into the right flank of each mouse. After tumour formation, mice in different groups were intraperitoneally injected (i.p.) with the following drugs: ADM (5 mg/kg, once daily), Epitalon (10 mg/kg, once daily, Selleck, USA), Chaetocin (2 mg/kg, once daily, Selleck, USA), and a combination of Epitalon (10 mg/kg, once daily) and Chaetocin (2 mg/kg, once daily). Tumour length (L) and width (W) were measured daily using callipers. The tumour volume was calculated using the following formula: V = (L×W²)/2. Mice were euthanised in accordance with ethical guidelines if an individual tumor grew beyond 20 mm in diameter or 2000 mm³ in volume, became ulcerated, or caused significant distress.

**20.Statistical analysis**

All data are presented as mean ± standard deviation (SD). For comparisons between two groups, an unpaired Student’s t-test was used for normally distributed data, whereas the Mann-Whitney U test was applied for non-normally distributed data. One-way analysis of variance followed by Tukey’s post-hoc test were used for multiple group comparisons. Receiver operating characteristic (ROC) curves were used to determine the cutoff values for plasma LCP2 levels in patients with R/R NKTCL. Correlations were analysed using Spearman’s correlation coefficients based on data normality.

Multiple comparison correction was performed using the Bonferroni method to control the false positive rate at the α = 0.05 level, ensuring the reliability of statistical results. The corrected *P* values were used for determining statistical significance (corrected *P* < 0.05). All statistical analyses were performed using GraphPad Prism 8.0 software.

**References**

1. Zhang X, Fu X, Dong M, Yang Z, Wu S, Ma M, et al. Conserved cell populations in doxorubicin-resistant human nasal natural killer/T cell lymphoma cell line: super multidrug resistant cells? Cancer Cell Int. 2018;18:150.

2. Zhang Y, Qian S, Wen Q, Lei Y, Ge J, Kong X, et al. SUV39H1 is a prognosis and immune microenvironment-related biomarker in diffuse large B-cell lymphoma. Clin Transl Oncol. 2023;25(8):2438-50.

3. Li H, Song W, Wu J, Shi Z, Gao Y, Li J, et al. CAR-T cells targeting CD38 and LMP1 exhibit robust antitumour activity against NK/T cell lymphoma. BMC Med. 2023;21(1):330.

**Supplementary Figures**


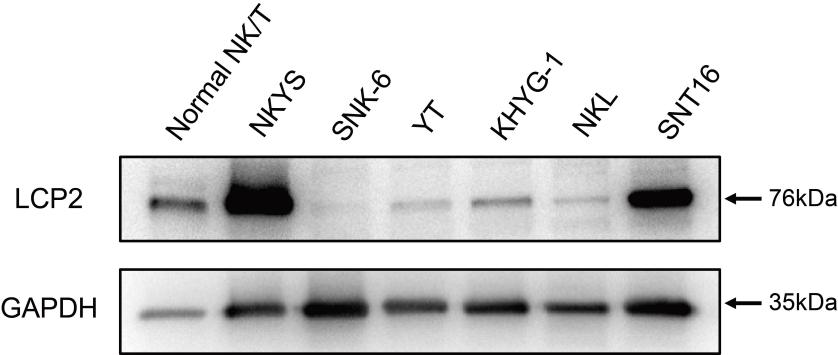


**Fig. S1**. **Expression of LCP2 in normal NK/T cells and NKTCL cell lines by WB (including NKYS, SNK-6, YT, KHYG-1, NKL, SNT16)**

**
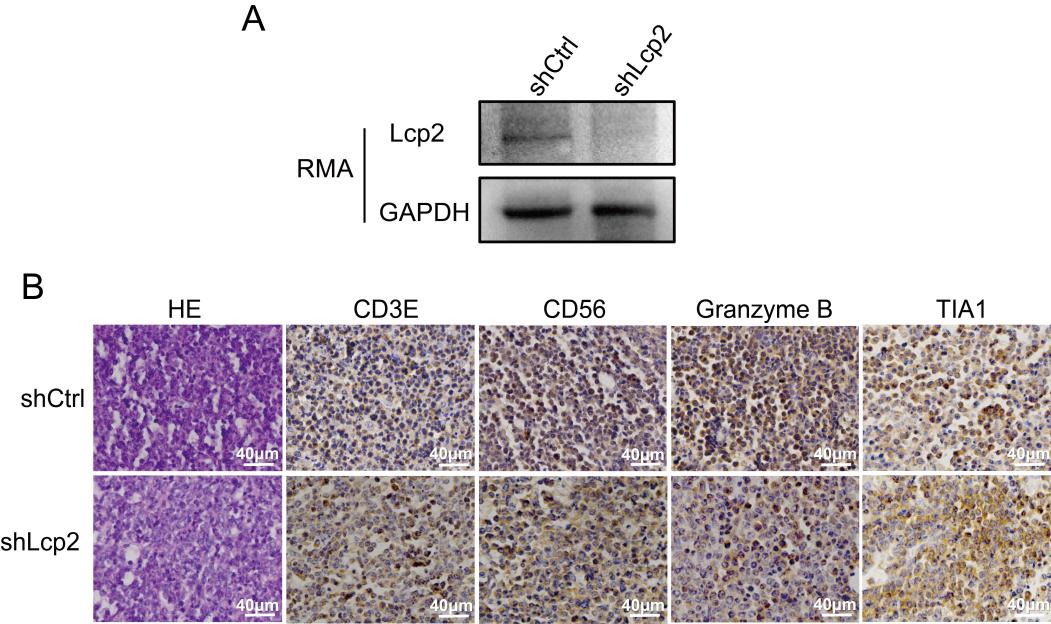
**

**Fig. S2 Construction of RMA-shLcp2 cells and HE/IHC staining in mouse NKTCL tumor tissues**

1. Lcp2 expression was examined on RMA-shLcp2 and RMA-shCtrl cells by WB;
   B. HE and IHC staining (CD3E, CD56, Granzyme B, TIA1) for RMA cells in tumour-bearing mouse models.


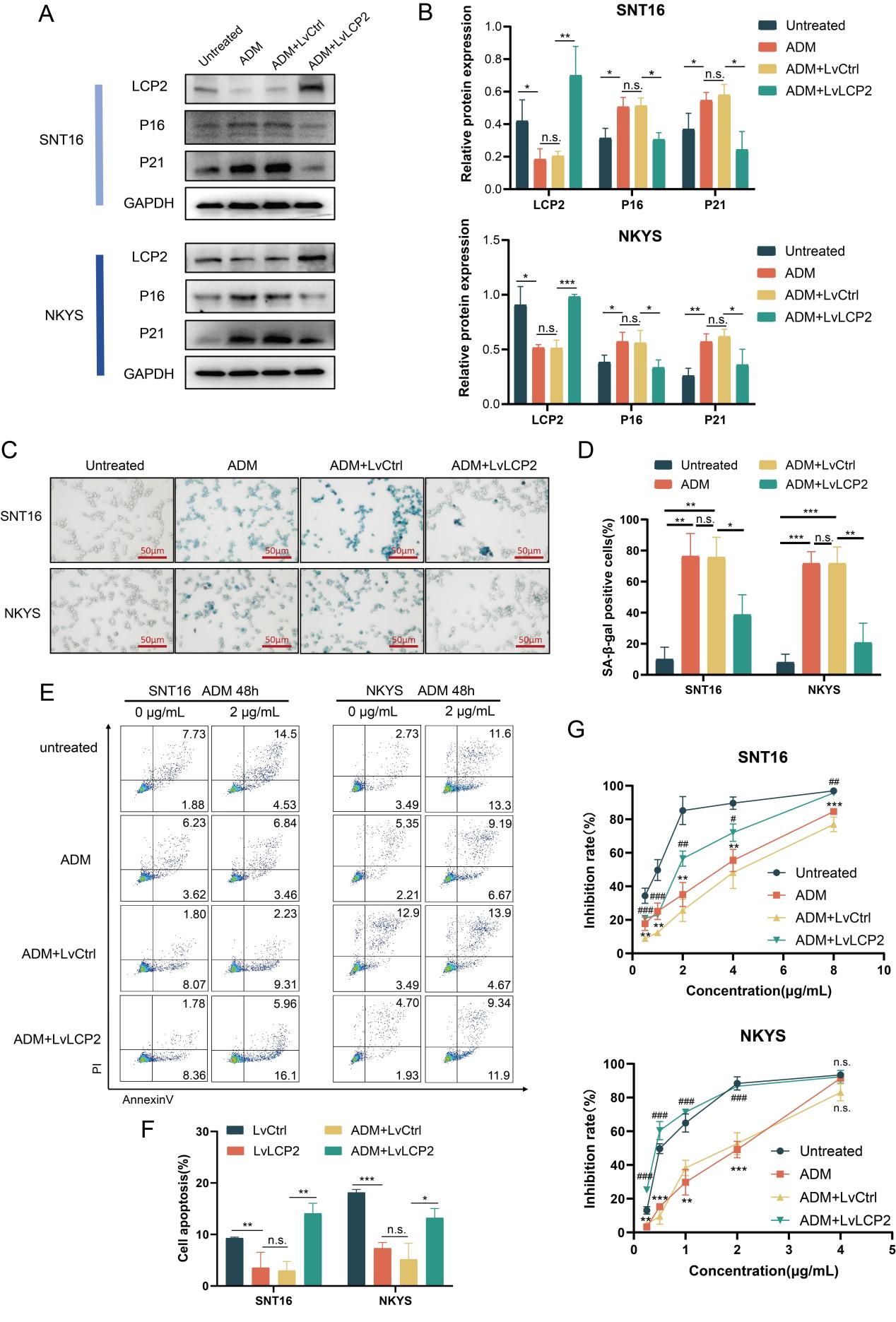


**Fig. S3 LCP2 overexpression reduces senescence-related chemoresistance in NKTCL-ADM cells.**

1. LCP2 expression was examined in NKTCL-Untreated, NKTCL-ADM, ADM-LvCtrl, and ADM-LvLCP2 cell lines by WB;
2. Statistical analysis histogram of the relative protein expression levels from Fig.A (n=3 per group)；
3. SA-β-gal staining was performed to detect cellular senescence in NKTCL-Untreated, NKTCL-ADM, ADM-LvCtrl, and ADM-LvLCP2 cells;
4. Statistical analysis histogram of the positive rate of SA-β-gal staining from Fig.C (n=3 per group);
5. Flow cytometry was used to detect cell apoptosis in NKTCL-Untreated, NKTCL-ADM, ADM-LvCtrl, and ADM-LvLCP2 cells after ADM treatment;
6. Statistical analysis histogram of the cell apoptosis rate from Fig.E (n=3 per group);
7. Comparison of ADM inhibition rates among NKTCL-Untreated, NKTCL-ADM, ADM-LvCtrl, and ADM-LvLCP2 cells by CCK-8 assay (n=3 per group);

**P* < 0.05, ***P* < 0.01, ****P* < 0.001 (NKTCL-ADM vs. NKTCL-Untreated); ^#^*P* < 0.05, ^##^*P* < 0.01, ^###^*P* < 0.001 (ADM-LvLCP2 group vs. ADM-LvCtrl group); n.s. = no significance; ADM: adriamycin.

**
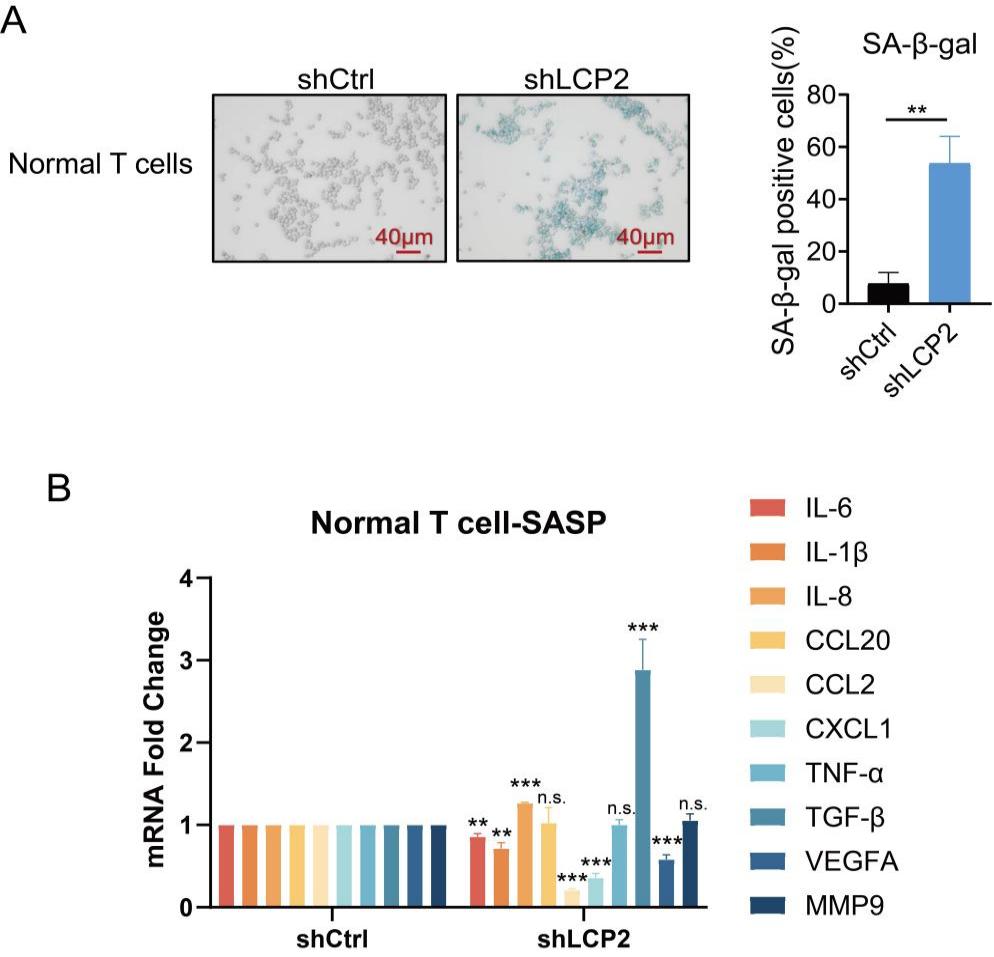
**

**Fig. S4 LCP2 downregulation facilitates senescence in normal T cells.**

A. SA-β-gal staining to detect cellular senescence in normal T cells-shCtrl and normal T cells-shLCP2 (n=3 per group);
B. RT-qPCR to detect SASP secretion in normal T cells-shCtrl and normal T cells-shLCP2 (n=3 per group).

***P* < 0.01, ****P* < 0.001, n.s. = no significance.


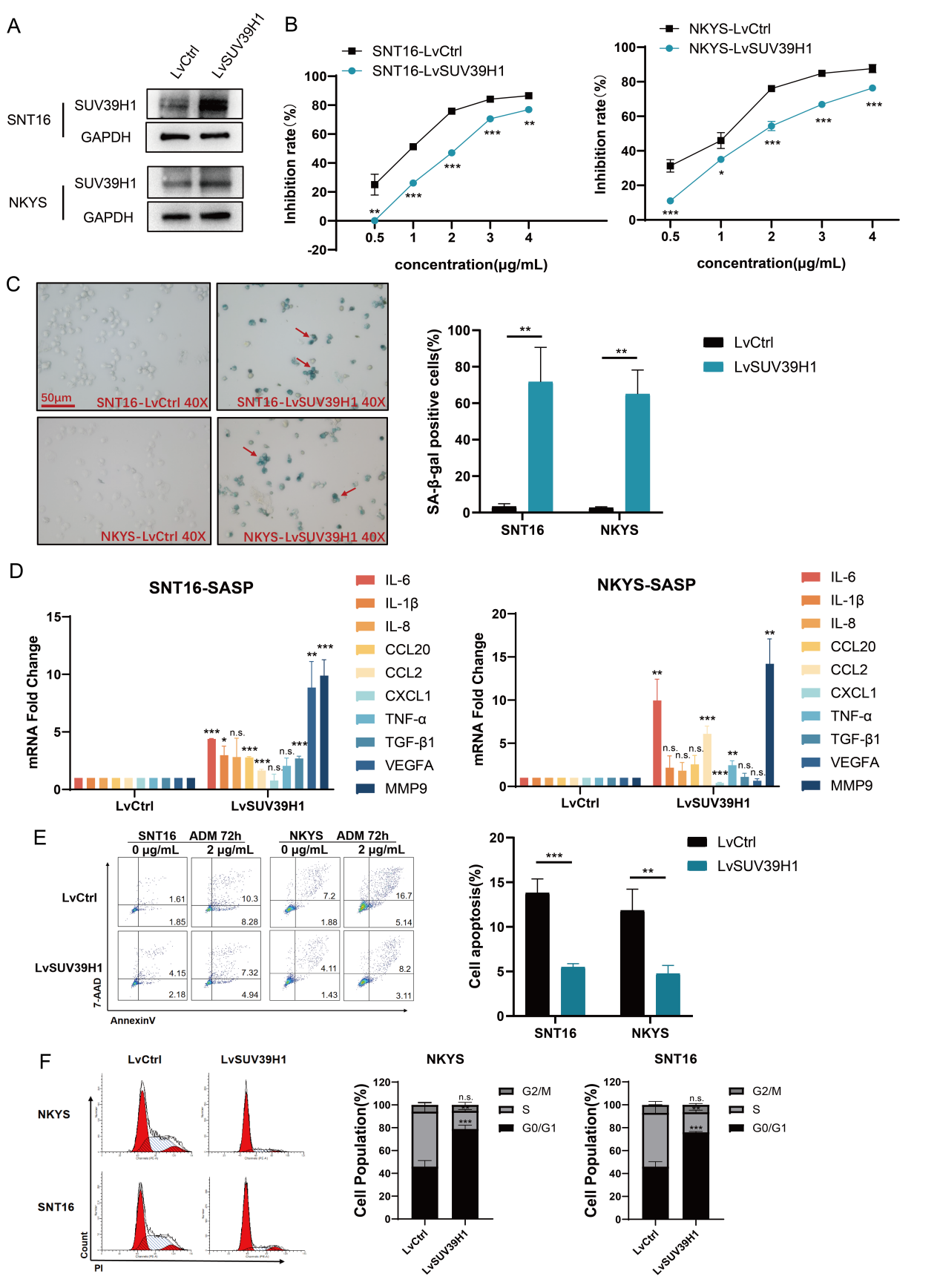


**Fig. S5 SUV39H1 overexpression facilitates senescence-related chemoresistance in NKTCL.**

1. Construction of NKTCL-LvSUV39H1 and NKTCL-LvCtrl cell lines;
   B. Comparison of ADM inhibition rates between NKTCL-LvSUV39H1 and NKTCL-LvCtrl cell lines by CCK-8 assay (n=3 per group);
   C. β-galactosidase staining to compare cellular senescence between NKTCL-LvSUV39H1 and NKTCL-LvCtrl cell lines, with bar graph showing SA-β-gal positive rate statistics (n=3 per group);
   D. RT-qPCR to detect SASP secretion in NKTCL-LvSUV39H1 and NKTCL-LvCtrl cell lines (n=3 per group);
2. Flow cytometry analysis of apoptosis in ADM-treated NKTCL-LvSUV39H1 and NKTCL-LvCtrl cell lines, with the quantitative bar graph (n=3 per group);
   F. Flow cytometry analysis of cell cycle arrest and the corresponding statistical bar graph showing cell cycle distribution in NKTCL-LvSUV39H1 and NKTCL-LvCtrl cell lines (n=3 per group).

**P* < 0.05, ***P* < 0.01, ****P* < 0.001, n.s. = no significance. ADM: adriamycin.


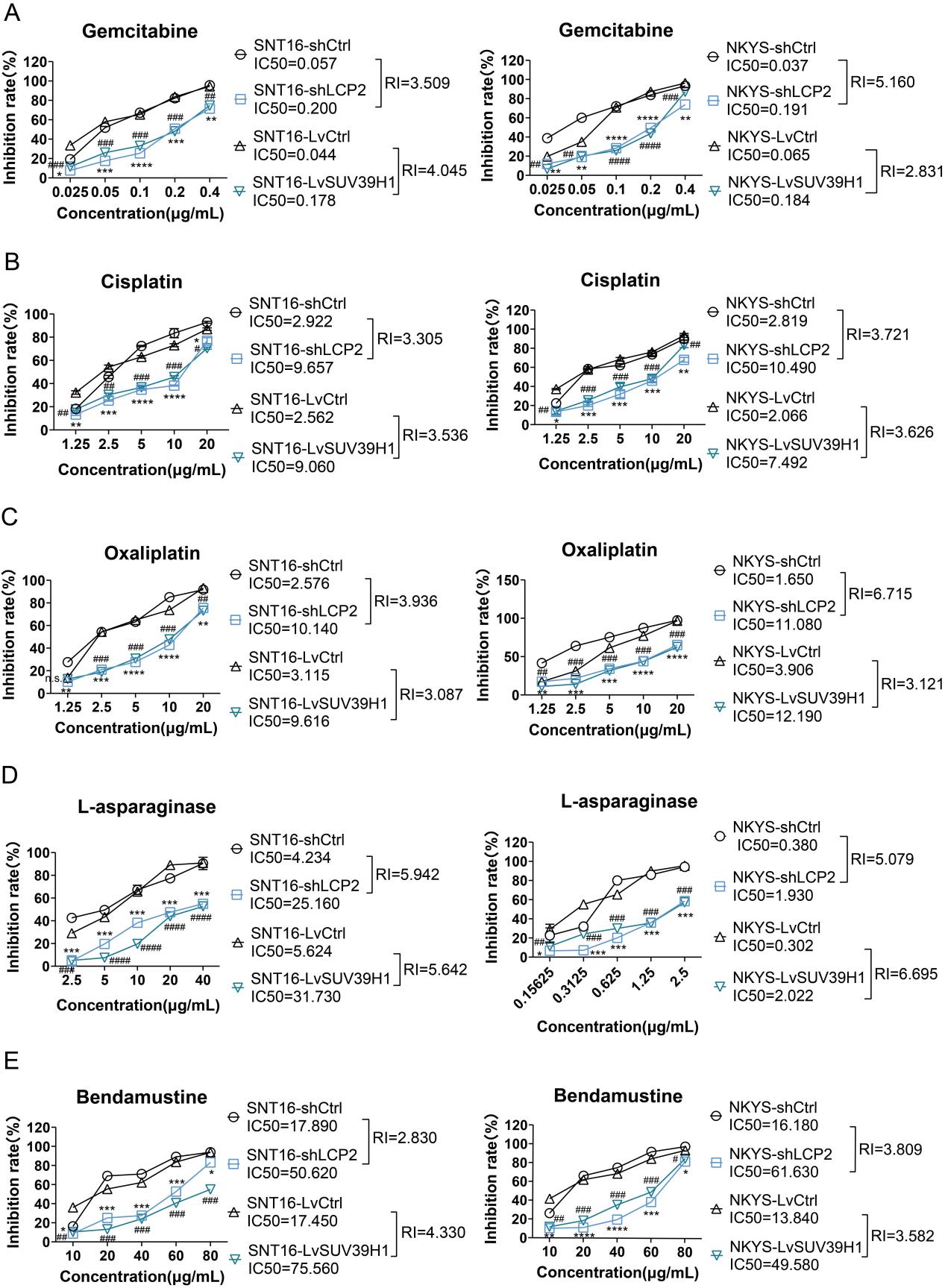


**Fig. S6 LCP2 knockdown or SUV39H1 overexpression increases multidrug resistance in NKTCL cells.**

A-E. The cell inhibition rates of NKTCL-shLCP2, NKTCL-LvSUV39H1, and their respective control counterparts after 72 h treatment with increasing concentrations of (A) gemcitabine, (B) cisplatin, (C) oxaliplatin, (D) L‑asparaginase, or (E) bendamustine, as measured by CCK‑8 assay (n=3 per group).

**P* < 0.05, ***P* < 0.01, ****P* < 0.001 (NKTCL-shLCP2 vs. NKTCL-shCtrl); ^#^*P* < 0.05, ^##^*P* < 0.01, ^###^*P* < 0.001 (NKTCL-LvSUV39H1 group vs. NKTCL-LvCtrl group); n.s. = no significance. RI, resistance index.
