# Supplementary material for: LCP2 mediates SUV39H1-driven cellular senescence-related chemoresistance in natural killer/T-cell lymphoma
Source: Cell Death Dis. 2026 May 28;17(1):662. doi: 10.1038/s41419-026-08897-6 (PMC13408427; doi:10.1038/s41419-026-08897-6)
Supplement: Supplementary file 2 — Supporting Information 2 [file 41419_2026_8897_MOESM2_ESM.docx]

Table S1. Primers for RT-qPCR.

| Primer name | Sequence(5’-3’) |
| --- | --- |
| IL6-F | CCTTCTCCACAAACATGTAACAAGA |
| IL6-R | GTGCCCATGCTACATTTGCC |
| IL-1β-F | AGCTCGCCAGTGAAATGATGG |
| IL-1β-R | GTCCTGGAAGGAGCACTTCAT |
| IL8-F | GAACTGCGCCTTGGTTTCTT |
| IL8-R | AAGACTAGGCCAGGCATCTC |
| CCL20-F | GGGTACTCAACACTGAGCAGA |
| CCL20-R | GGTGGAGTAGCAGCACTGAC |
| CCL2-F | CCTTCATTCCCCAAGGGCTC |
| CCL2-R | GGTTTGCTTGTCCAGGTGGT |
| CXCL1-F | AGGAGGCCCTGCCCTTATAG |
| CXCL1-R | TCCAGTAAAGGTAGCCCTTGTTT |
| TNFa-F | CACAGTGAAGTGCTGGCAAC |
| TNFa-R | ACATTGGGTCCCCCAGGATA |
| TGF-β1-F | TACCTGAACCCGTGTTGCTC |
| TGF-β1-R | CGGTAGTGAACCCGTTGATGT |
| VEGFA-F | ACATCACCATGCAGATTATGCG |
| VEGFA-R | CTCCAGGGCATTAGACAGCA |
| MMP9-F | TCTATGGTCCTCGCCCTGAA |
| MMP9-R | CATCGTCCACCGGACTCAAA |
| ALDH1A1-F | CATGGCAATGAGGCCACAAC |
| ALDH1A1-R | CTGAAGAGTGGTCAGGGTTCC |
| CD133-F | AGAAGGATGGCCAAAGGGTG |
| CD133-R | GCTCCGTAAGCTCCCTTGTT |
| NANOG-F | GGACACACGGGATGATGCTC |
| NANOG-R | TTCTCACCTGTGTGGGTTCG |
| CD34-F | TTCAGTACTTCAGCATTCCACGAT |
| CD34-R | ATTGTTTCCTGTTGCATTGAGTCC |
| SCAL-F | GCCAAGATGTTGCCTATGTGCTT |
| SCAL-R | CACAGAGACCACACCAACCA |
| KLF4-F | CATGGCAATGAGGCCACAAC |
| KLF4-R | CTGAAGAGTGGTCAGGGTTCC |
| ABCG2-F | AGAAGGATGGCCAAAGGGTG |
| ABCG2-R | GCTCCGTAAGCTCCCTTGTT |
| ABCC4-F | GGACACACGGGATGATGCTC |
| ABCC4-R | TTCTCACCTGTGTGGGTTCG |
| GAPDH-F | TTGCAACCGGGAAGGAAATG |
| GAPDH-R | TGGAATTTGCCATGGGTGGA |
| P62-F | CATTGCGGAGCCTCATCTCCT |
| P62-R | CCTCGCAGACGCTACACAA |
| CDKN2A(P16)-F | CCGAATAGTTACGGTCGGAGG |
| CDKN2A(P16)-R | AATCGGGGATGTCTGAGGGA |
| CDKN1A(P21)-F | CCGAAGTCAGTTCCTTGTGGA |
| CDKN1A(P21)-R | GGAAGGTAGAGCTTGGGCAG |
| P53-F | AAGTCTAGAGCCACCGTCCA |
| P53-R | ACCATCGCTATCTGAGCAGC |
| LCP2-F | CTTCACACGCAAACCCCAAG |
| LCP2-R | TCTTCAAAGGACGACCAGCC |
| SUV39H1-F | TGATGAGGGGCGGATTGAAC |
| SUV39H1-R | GCCTTCTGCACCAGGTAGTT |
| Lcp2-F | GCAAATCGCCGAGCACTTAG |
| Lcp2-R | GAACTGTTGCAGGCCCAAAG |
| p16(INK4a)-F | CGAACTCGAGGAGAGCCATC |
| p16(INK4a)-R | TACGTGAACGTTGCCCATCA |
| p21(Cip1)-F | TAAGGACGTCCCACTTTGCC |
| p21(Cip1)-R | CTGAGGATCACCCCCAGGTA |

Table S2. Information of antibodies.

| Protein name | Application | Corporation | Product code |
| --- | --- | --- | --- |
| P-gp | WB CCTTCTCCACAAACATGTAACAAGA | Proteintech | 22336-1-AP |
| ABCG2 | WB | [Cell Signalling Technology](https://www.so.com/link?m=env02NZCxupQcz/c8JrypvYgEelZ4A5M+GnHcPH7YUz3LLhplrpAOGmevwCTDu2xgu7xqc7MR6e4bSRHrLj96rrTBZEeLsG8vAxA8rQ0+sfSINn5iHpg7bk5IEOQ9UEh2QyapxvgEr7HsJXu/s1oV6A==) | 42078T |
| NANOG | WB | [Cell Signalling Technology](https://www.so.com/link?m=env02NZCxupQcz/c8JrypvYgEelZ4A5M+GnHcPH7YUz3LLhplrpAOGmevwCTDu2xgu7xqc7MR6e4bSRHrLj96rrTBZEeLsG8vAxA8rQ0+sfSINn5iHpg7bk5IEOQ9UEh2QyapxvgEr7HsJXu/s1oV6A==) | 4903T |
| SUV39H1 | WB/IP | Proteintech | 10574-1-AP |
| H3K9me3 | WB | Proteintech | 39285 |
| β-tubulin | WB | [Cell Signalling Technology](https://www.so.com/link?m=env02NZCxupQcz/c8JrypvYgEelZ4A5M+GnHcPH7YUz3LLhplrpAOGmevwCTDu2xgu7xqc7MR6e4bSRHrLj96rrTBZEeLsG8vAxA8rQ0+sfSINn5iHpg7bk5IEOQ9UEh2QyapxvgEr7HsJXu/s1oV6A==) | 2128T |
| GAPDH | WB | Proteintech | 60004-1-Ig |
| SLP76(LCP2) | WB/IHC/IP/IF | Proteintech/Cell Signaling Technology | 12728-1-AP/4958T |
| β-galactosidase | WB/IF | Proteintech | 66586-1-lg |
| LaminA/C | WB/IP/IHC | Proteintech | 10298-1-AP |
| p-LaminA/C(S51ph) | WB/IP/IHC | PTM BIO | CDPTM-1301 |
| MRE11 | WB | Proteintech | 10744-1-AP |
| H2A.X | WB | Proteintech | 68888-1-Ig |
| p-H2A.X | WB | Proteintech | 83307-2-RR |
| TERF2 | WB | Proteintech | 66893-1-Ig |
| IQGAP2 | WB/IP | Santa Cruz | sc-17835 |
| IQGAP1 | WB | Proteintech | 22167-1-AP |
| Cleaved caspase 3 | WB | Proteintech | 25128-1-AP |
| P21 | WB | Proteintech | 10355-1-AP/28248-1-AP |
| P16 | WB | Proteintech | 60626-1-Ig/28416-1-AP |
| CD3 | IHC | Proteintech | 17617-1-AP |
| CD56 | IHC | Proteintech | 14255-1-AP |
| Granzyme B | IHC | Proteintech | 13588-1-AP |
| TIA-1 | IHC | Proteintech | 12133-2-AP |
| CD3 | FC | BD | 347344 |
| CD4 | FC | BD | 566908 |
| CD8 | FC | BD | 348793 |
| CD25 | FC | BD | 557138 |
| CD127 | FC | Invitrogen | 404-1271-82 |
| FITC anti-human CD69 | FC | Biolegend | 310903 |
| FITC anti-human CD279 (PD-1) | FC | Biolegend | 379205 |
| PerCP/Cyanine5.5 anti-human CD223 (LAG-3) | FC | Biolegend | 369311 |
| PE anti-human CD366 (Tim-3) | FC | Biolegend | 364805 |
| PE anti-human IFN-γ | FC | Biolegend | 506506 |
| IFN-γ | IF | Absin | abs119966 |
| CD8 | IF | Cell Signalling Technology | 98941S |
| CD3 | IF | Cell Signalling Technology | 78588 |
| PD-1 | IF | Cell Signalling Technology | 84651 |
| TIM3 | IF | Abcam | ab241332 |

WB: Western Blotting; IHC: Immunohistochemistry; IP: Immunoprecipitation.; FC: Flow Cytometry; IF:Immunofluorescence.
