# Supplementary material for: LCP2 mediates SUV39H1-driven cellular senescence-related chemoresistance in natural killer/T-cell lymphoma
Source: Cell Death Dis. 2026 May 28;17(1):662. doi: 10.1038/s41419-026-08897-6 (PMC13408427; doi:10.1038/s41419-026-08897-6)
Supplement: Supplementary file 4 — Original Data File of Western Blotting [file 41419_2026_8897_MOESM4_ESM.pdf]

Figure 1E

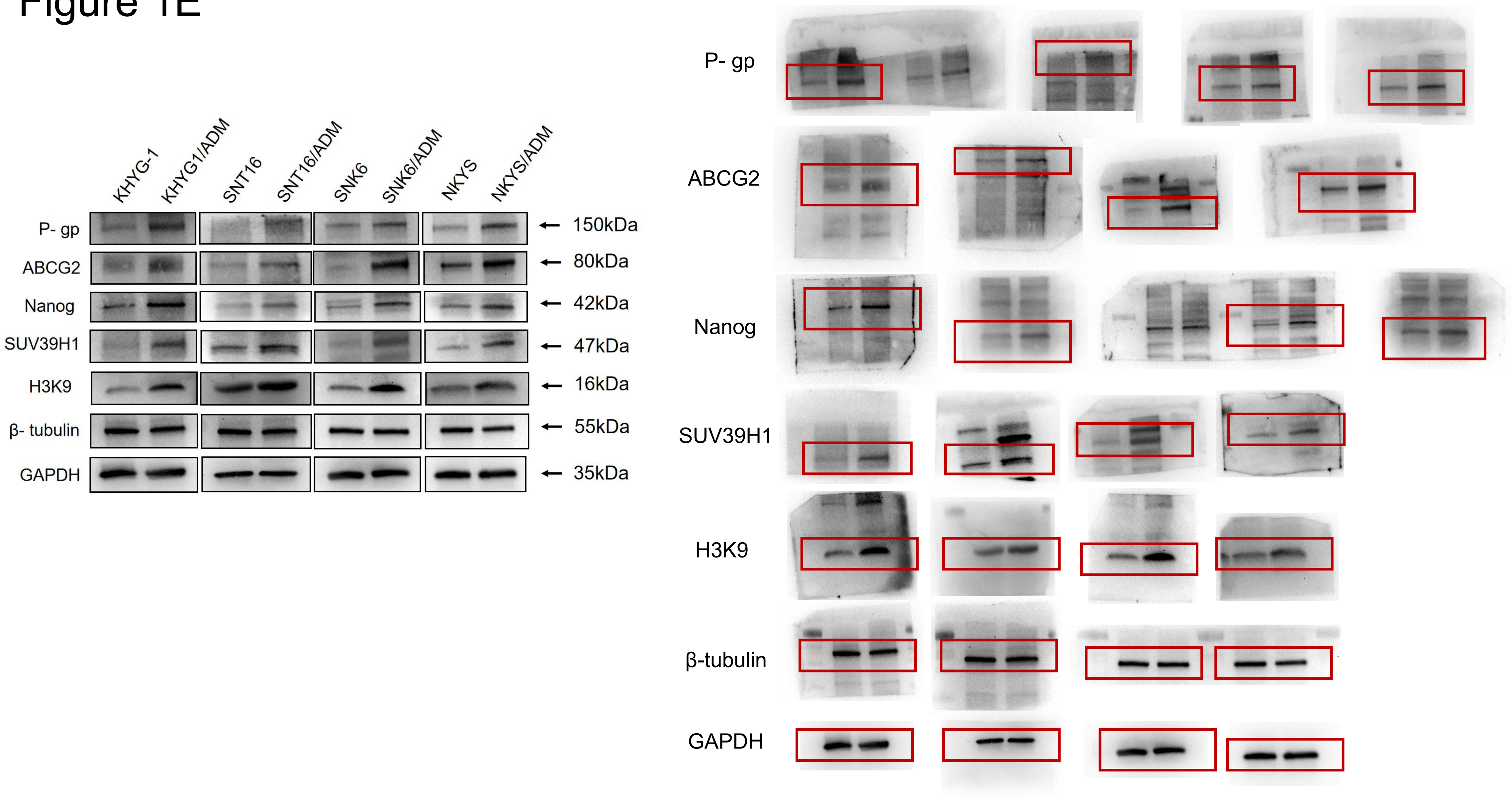
KHYG-1
KHYG1/ADM
SNT16
SNT16/ADM
SNK6
SNK6/ADM
NKYS
NKYS/ADM
P- gp
ABCG2
Nanog
SUV39H1
H3K9
β- tubulin
GAPDH
150kDa
80kDa
42kDa
47kDa
16kDa
55kDa
35kDa
P- gp
ABCG2
Nanog
SUV39H1
H3K9
β-tubulin
GAPDH

Figure 2F

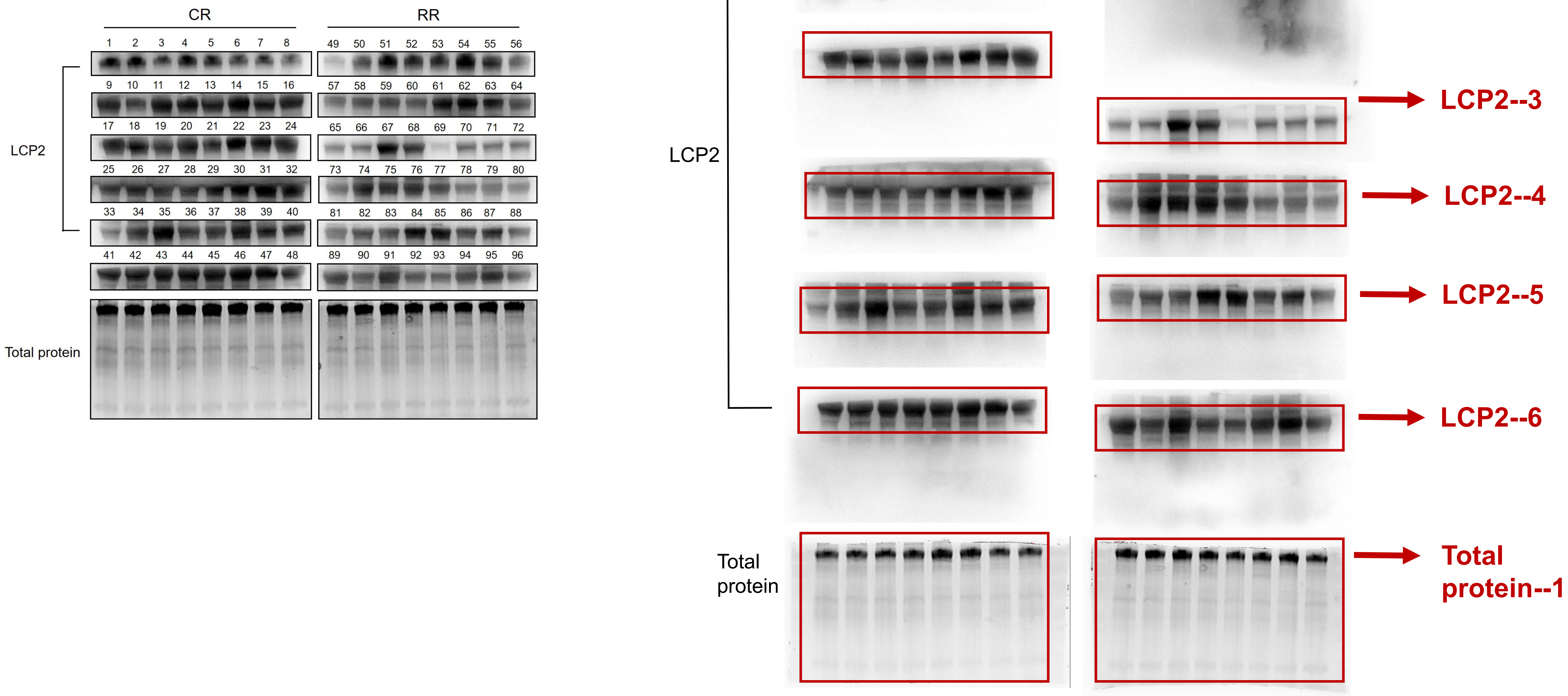
CR
RR
1 2 3 4 5 6 7 8
49 50 51 52 53 54 55 56
9 10 11 12 13 14 15 16
57 58 59 60 61 62 63 64
17 18 19 20 21 22 23 24
65 66 67 68 69 70 71 72
25 26 27 28 29 30 31 32
73 74 75 76 77 78 79 80
33 34 35 36 37 38 39 40
81 82 83 84 85 86 87 88
41 42 43 44 45 46 47 48
89 90 91 92 93 94 95 96
LCP2
Total protein
LCP2
Total protein
LCP2--1
LCP2--2
LCP2--3
LCP2--4
LCP2--5
LCP2--6
Total protein--1

Figure 2F Total protein

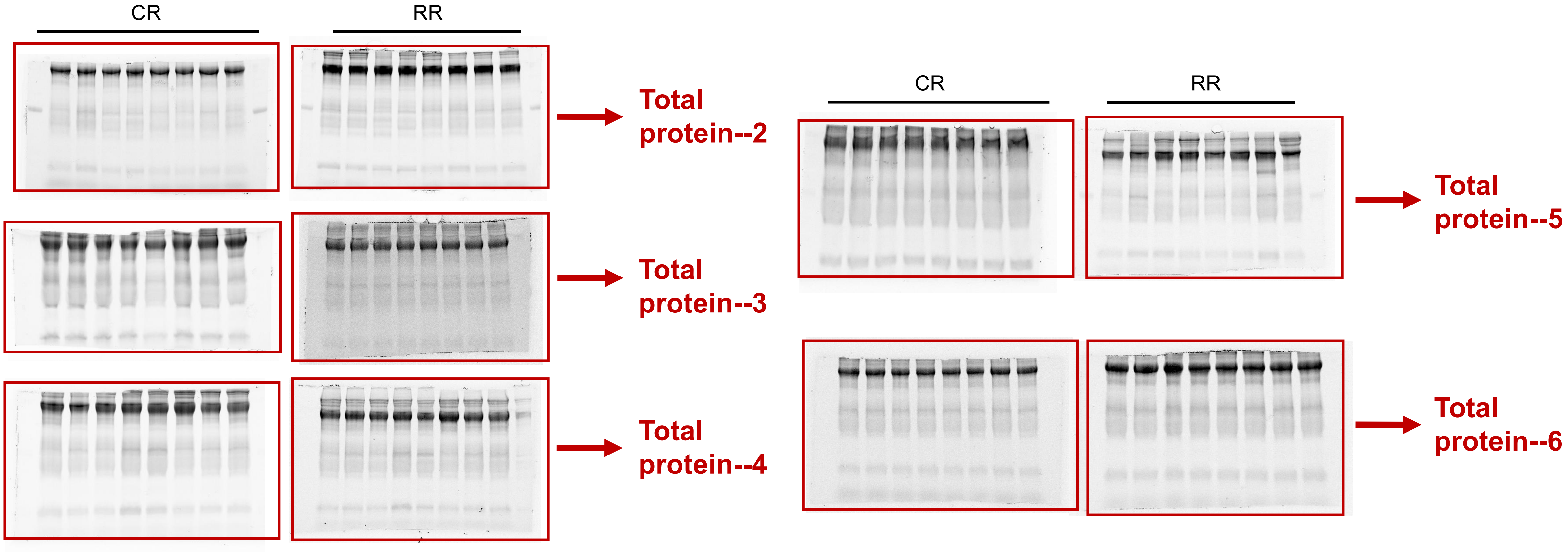

Figure 2L

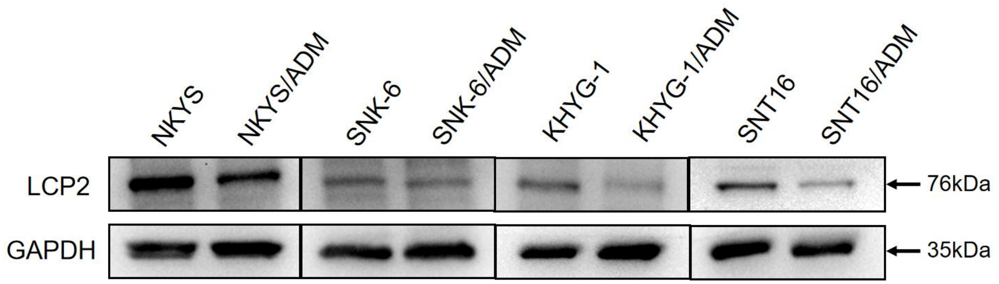

NKYS
NKYS/ADM
SNK-6
SNK-6/ADM
KHYG-1
KHYG-1/ADM
SNT16
SNT16/ADM
LCP2
GAPDH
76kDa
35kDa

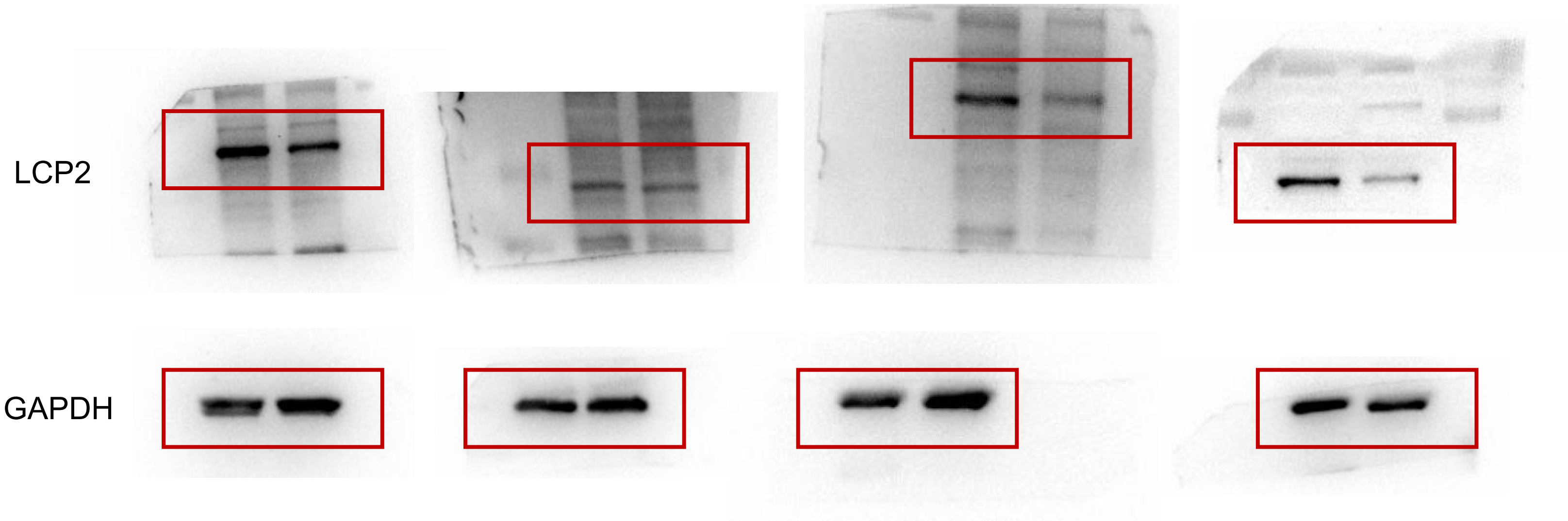

LCP2
GAPDH

Figure 3A

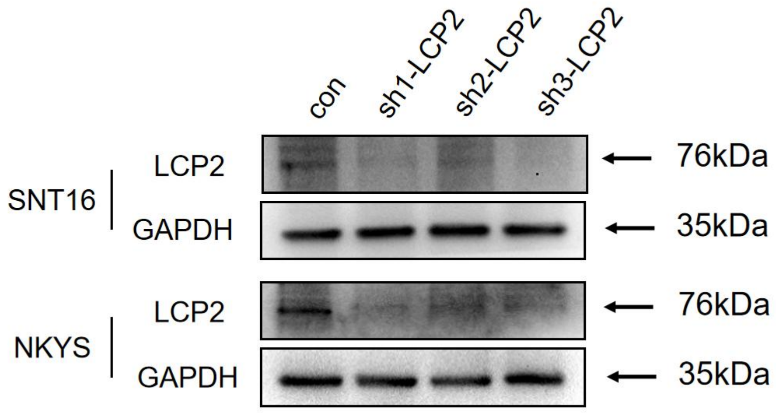
con
sh1-LCP2
sh2-LCP2
sh3-LCP2
SNT16
LCP2
GAPDH
NKYS
LCP2
GAPDH
76kDa
35kDa
76kDa
35kDa

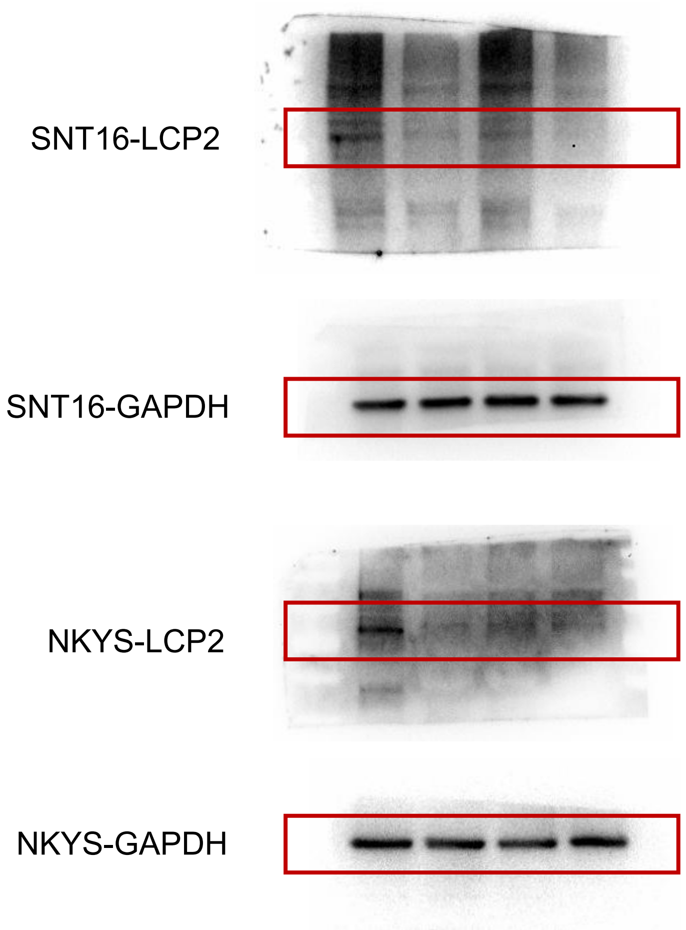
SNT16-LCP2
SNT16-GAPDH
NKYS-LCP2
NKYS-GAPDH

Figure 4D

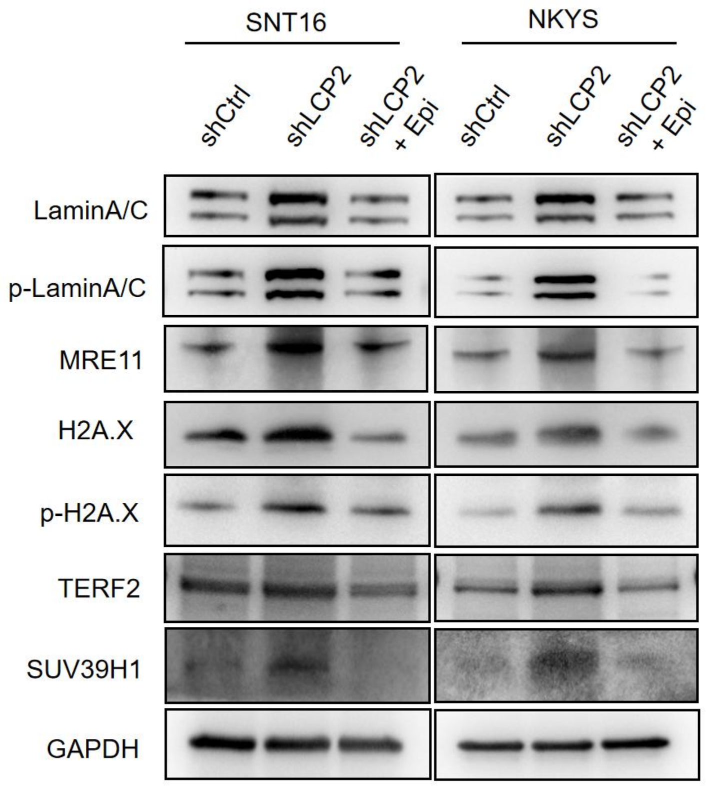
SNT16
NKYS
shCtrl
shLCP2
shLCP2 + Epi
shCtrl
shLCP2
shLCP2 + Epi
LaminA/C
p-LaminA/C
MRE11
H2A.X
p-H2A.X
TERF2
SUV39H1
GAPDH

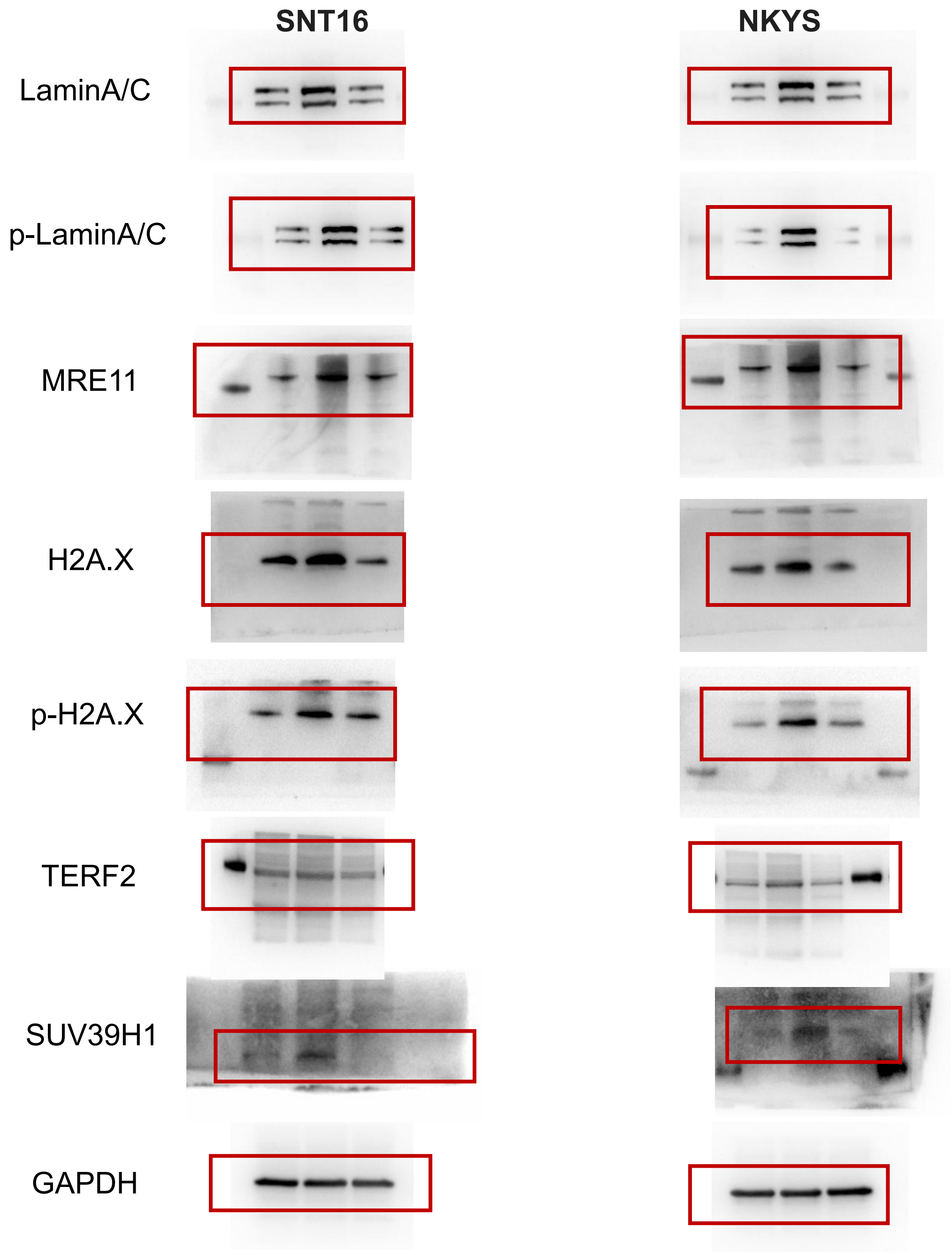
SNT16
NKYS
LaminA/C
p-LaminA/C
MRE11
H2A.X
p-H2A.X
TERF2
SUV39H1
GAPDH

Figure 4E

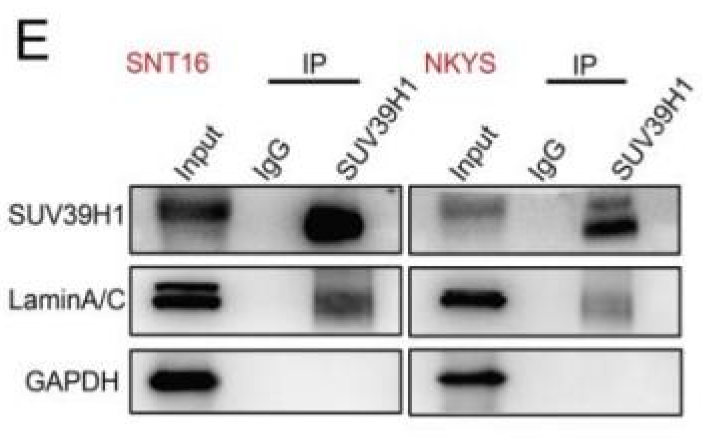

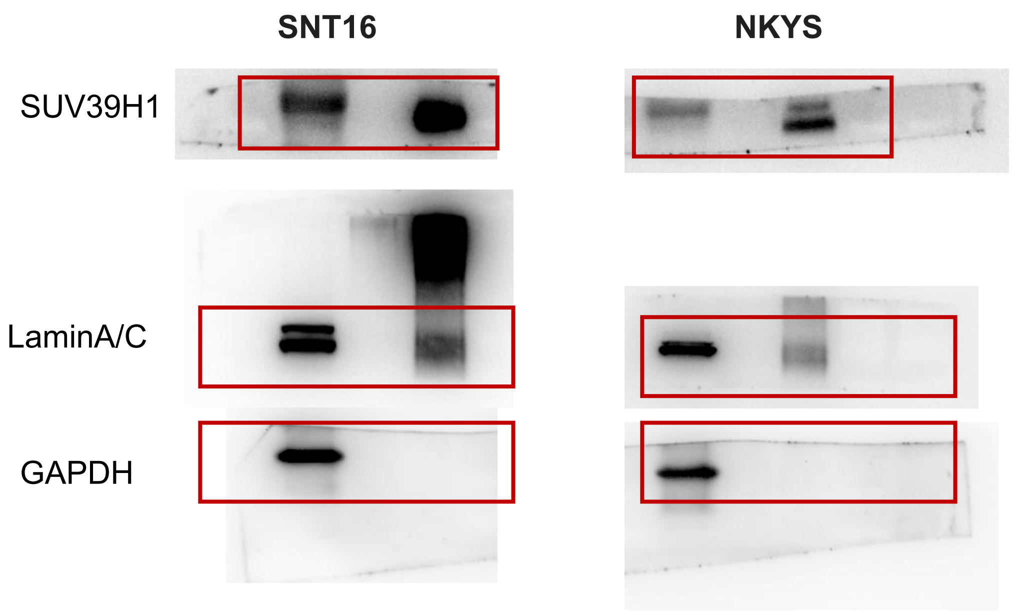

Figure 4F

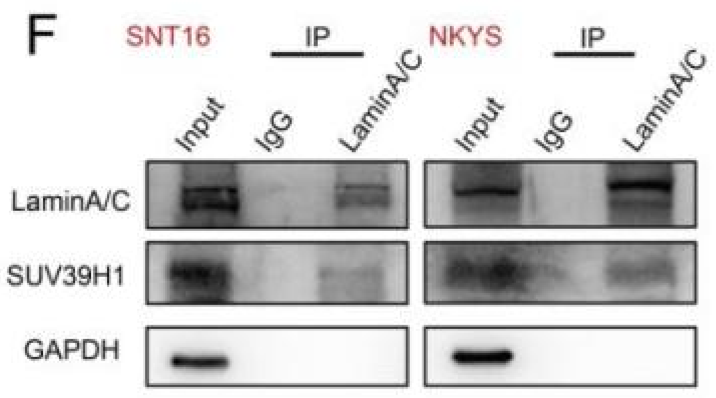

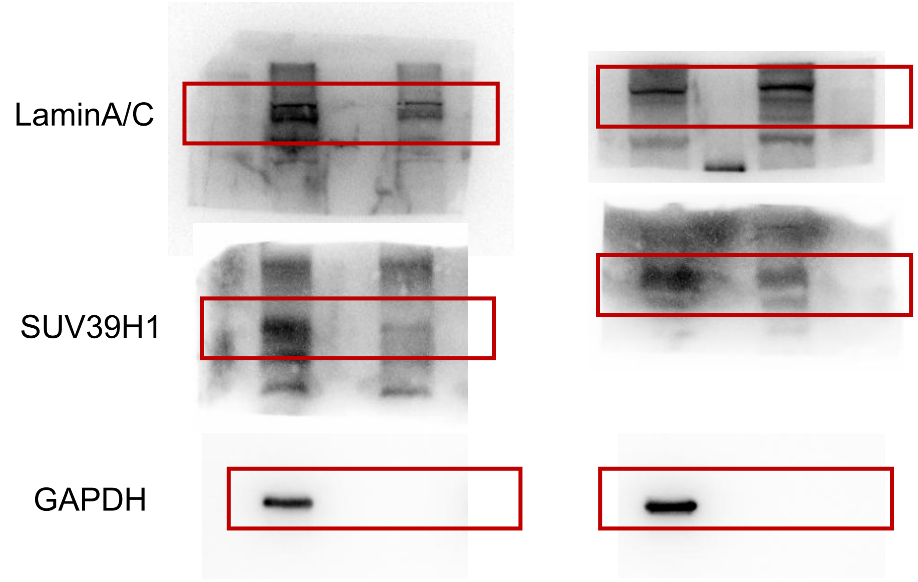

Figure 4H

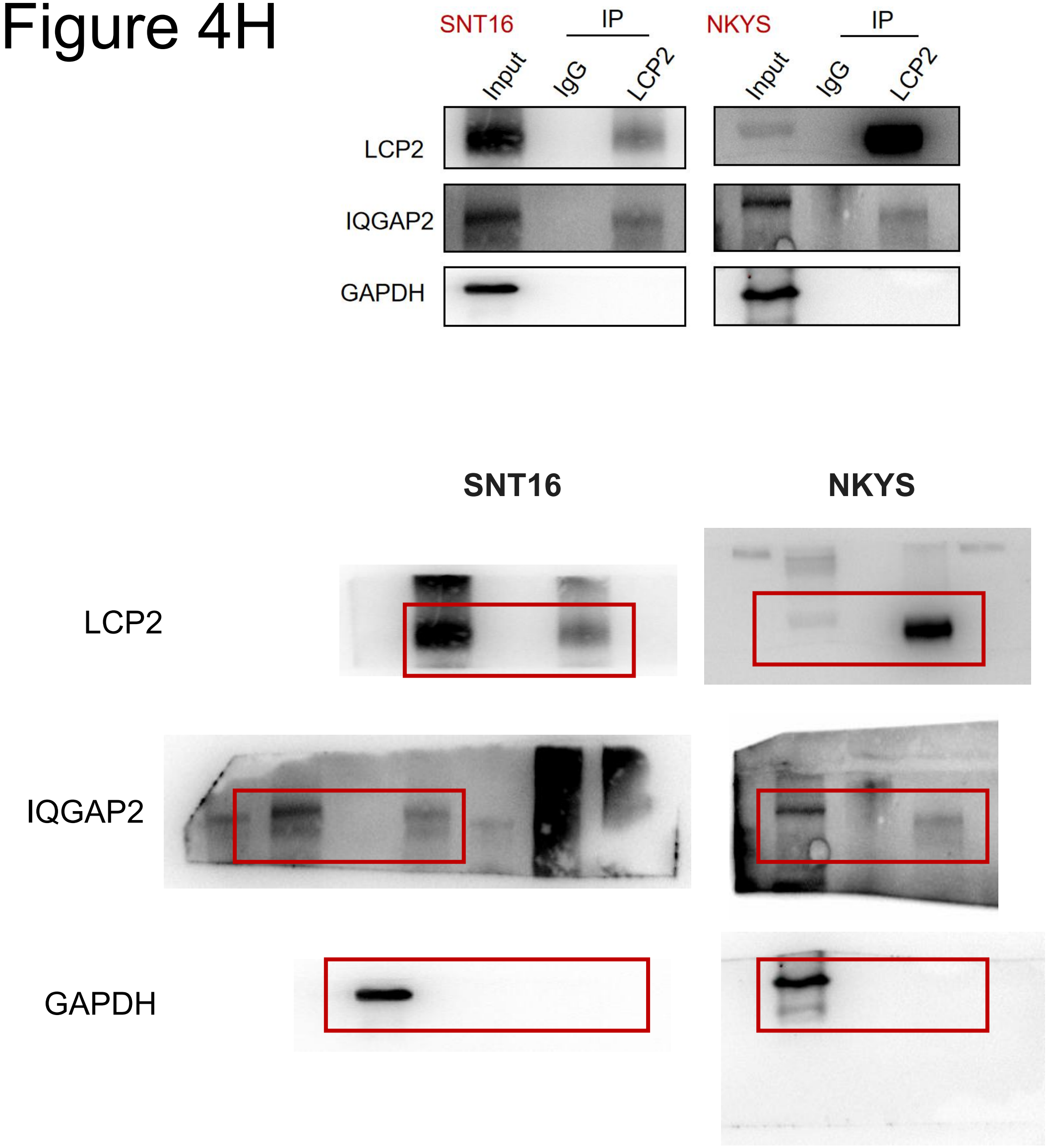

Figure 4I

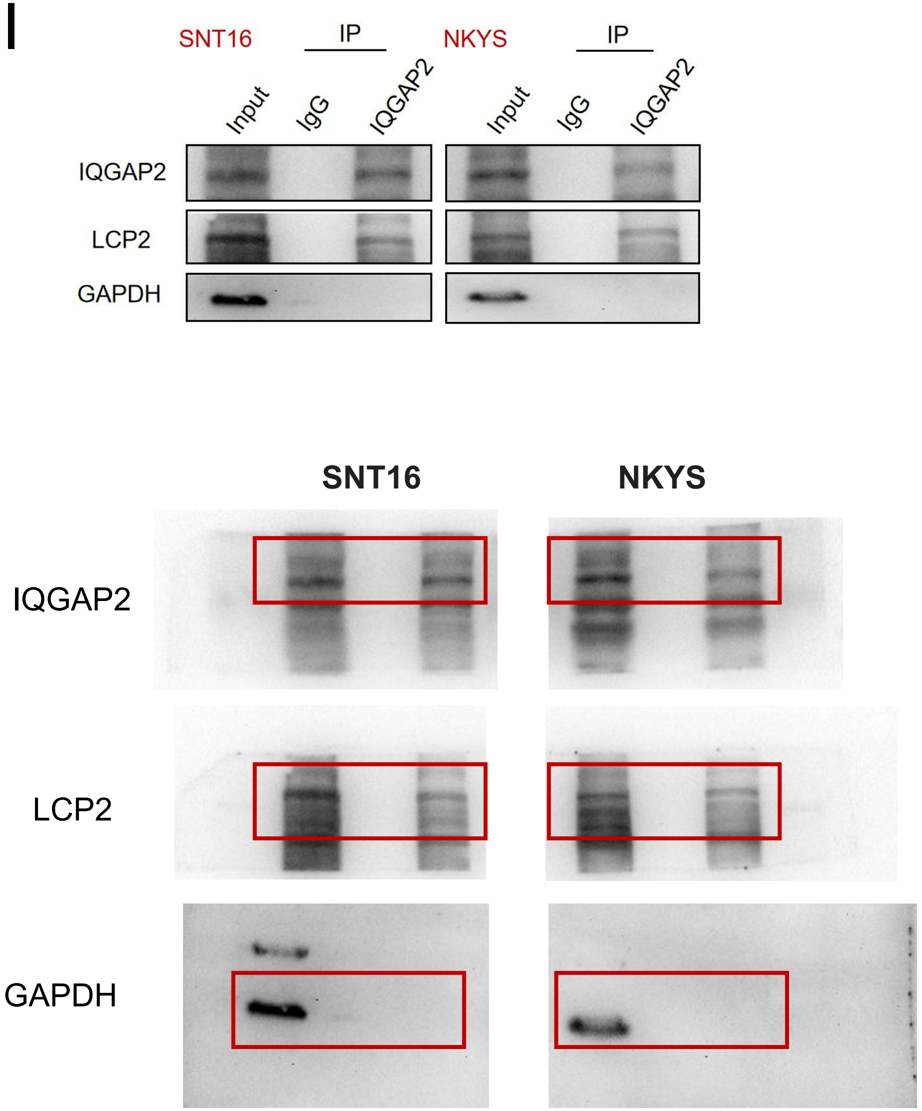

Figure 4J

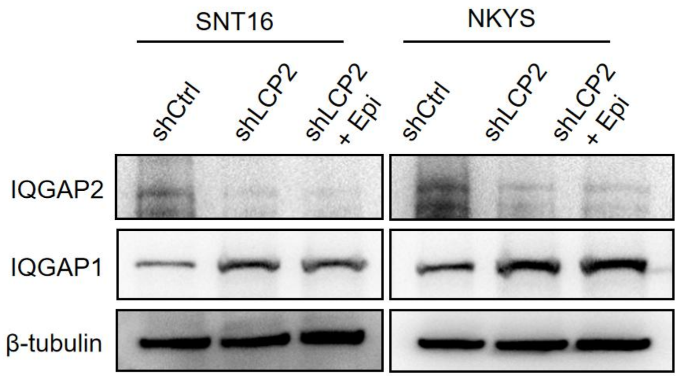

SNT16
NKYS
shCtrl
shLCP2
shLCP2 + Epi
shCtrl
shLCP2
shLCP2 + Epi
IQGAP2
IQGAP1
β-tubulin

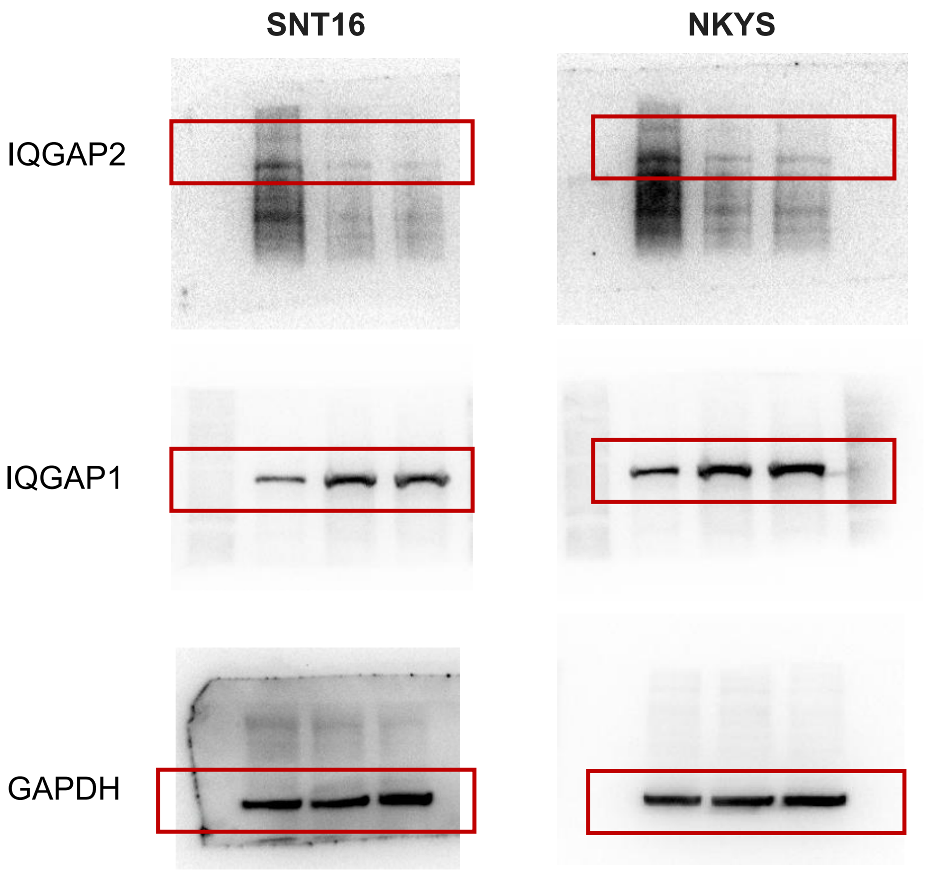

SNT16
NKYS
IQGAP2
IQGAP1
GAPDH

Figure 4K

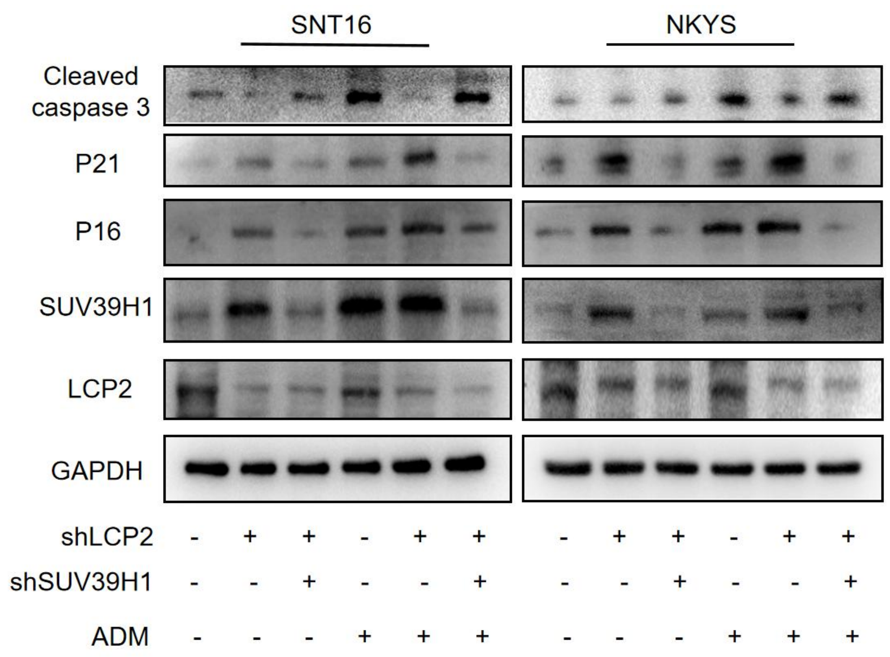

SNT16
NKYS
Cleaved caspase 3
P21
P16
SUV39H1
LCP2
GAPDH
shLCP2 - + + - + + - + + - + +
shSUV39H1 - - + - - + - - + - - +
ADM - - - + + + - - - + + +

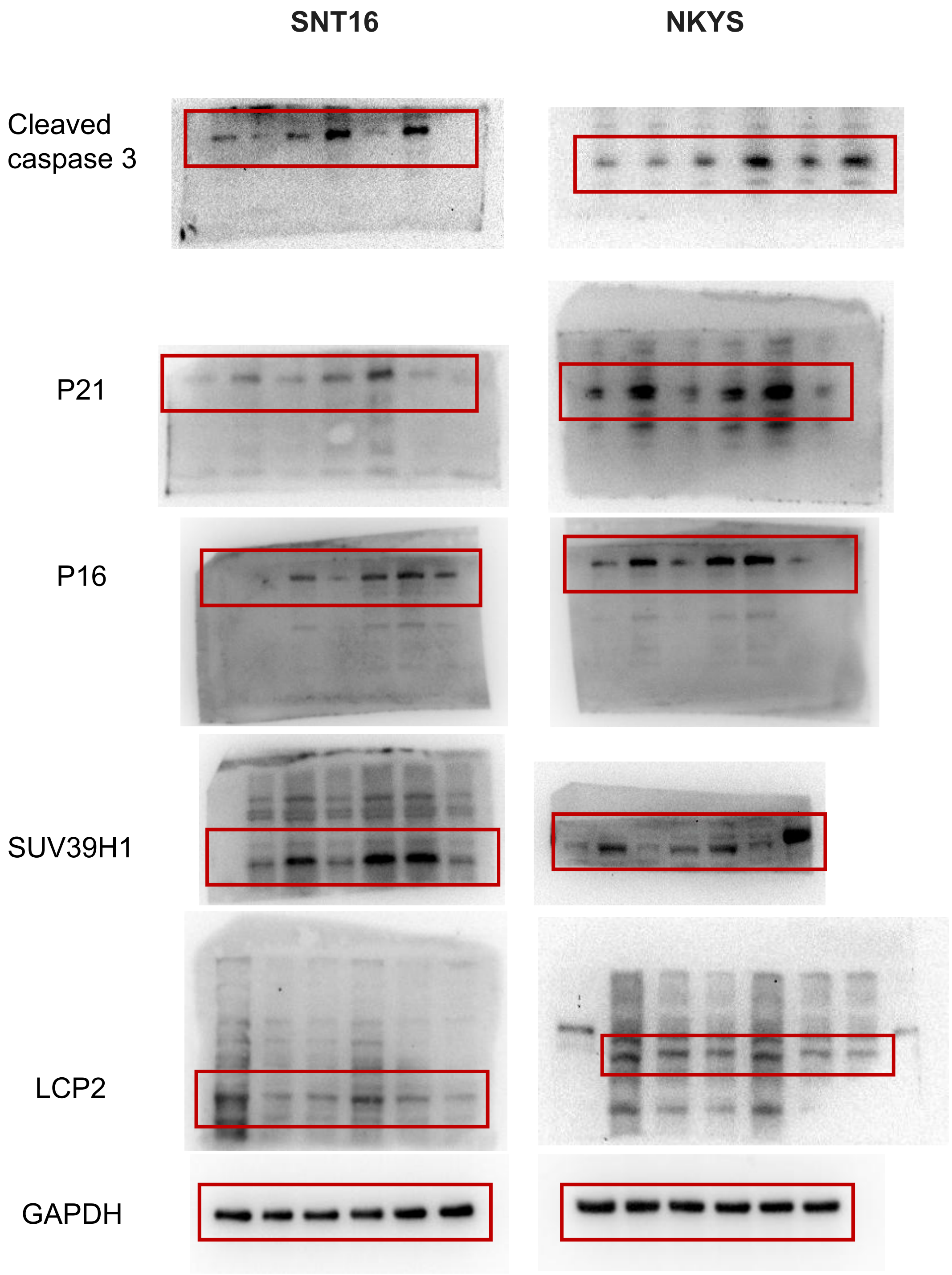

SNT16
NKYS
Cleaved caspase 3
P21
P16
SUV39H1
LCP2
GAPDH

Figure 6I

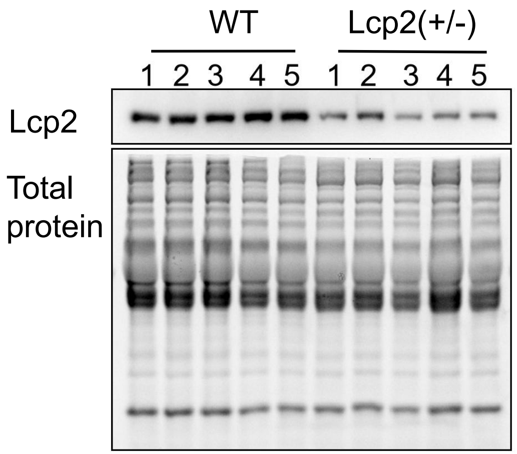

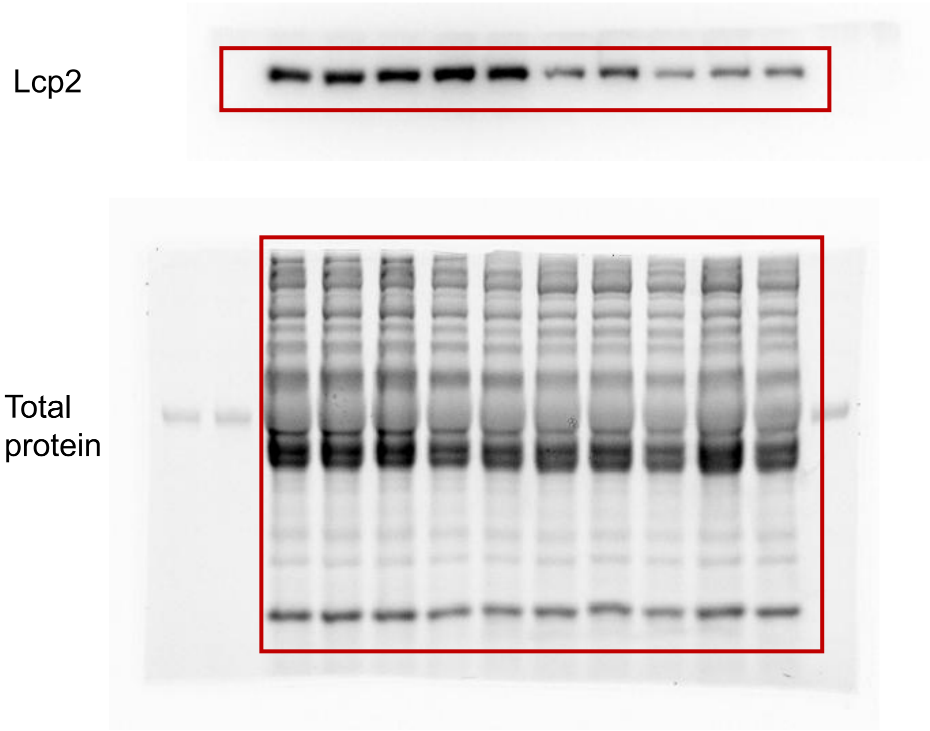

Figure 6G

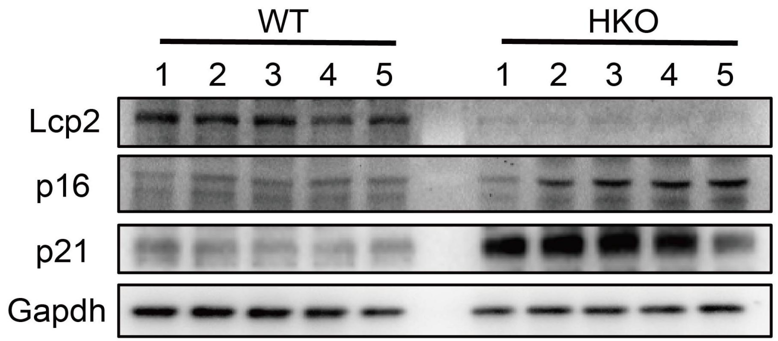

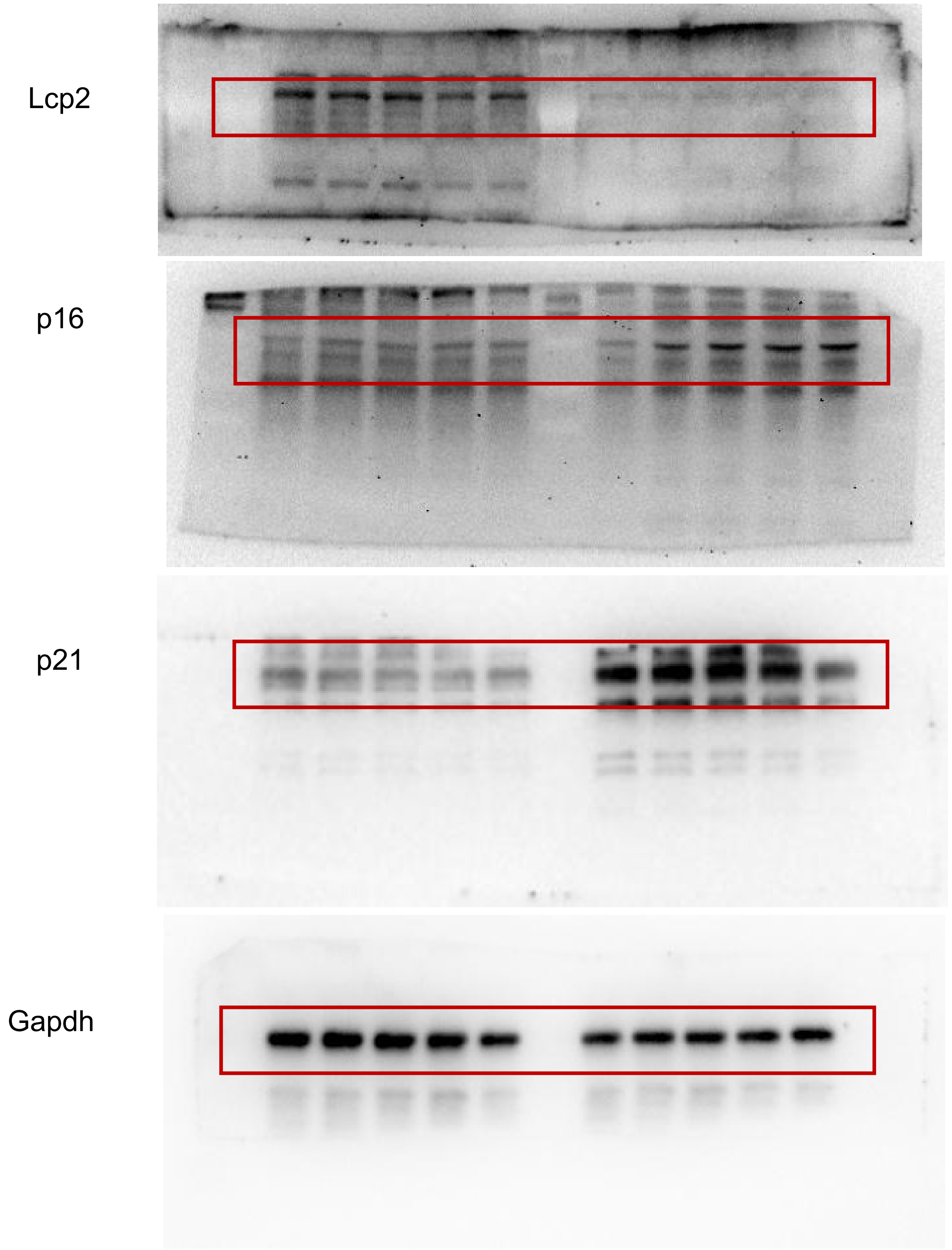

Figure 6O

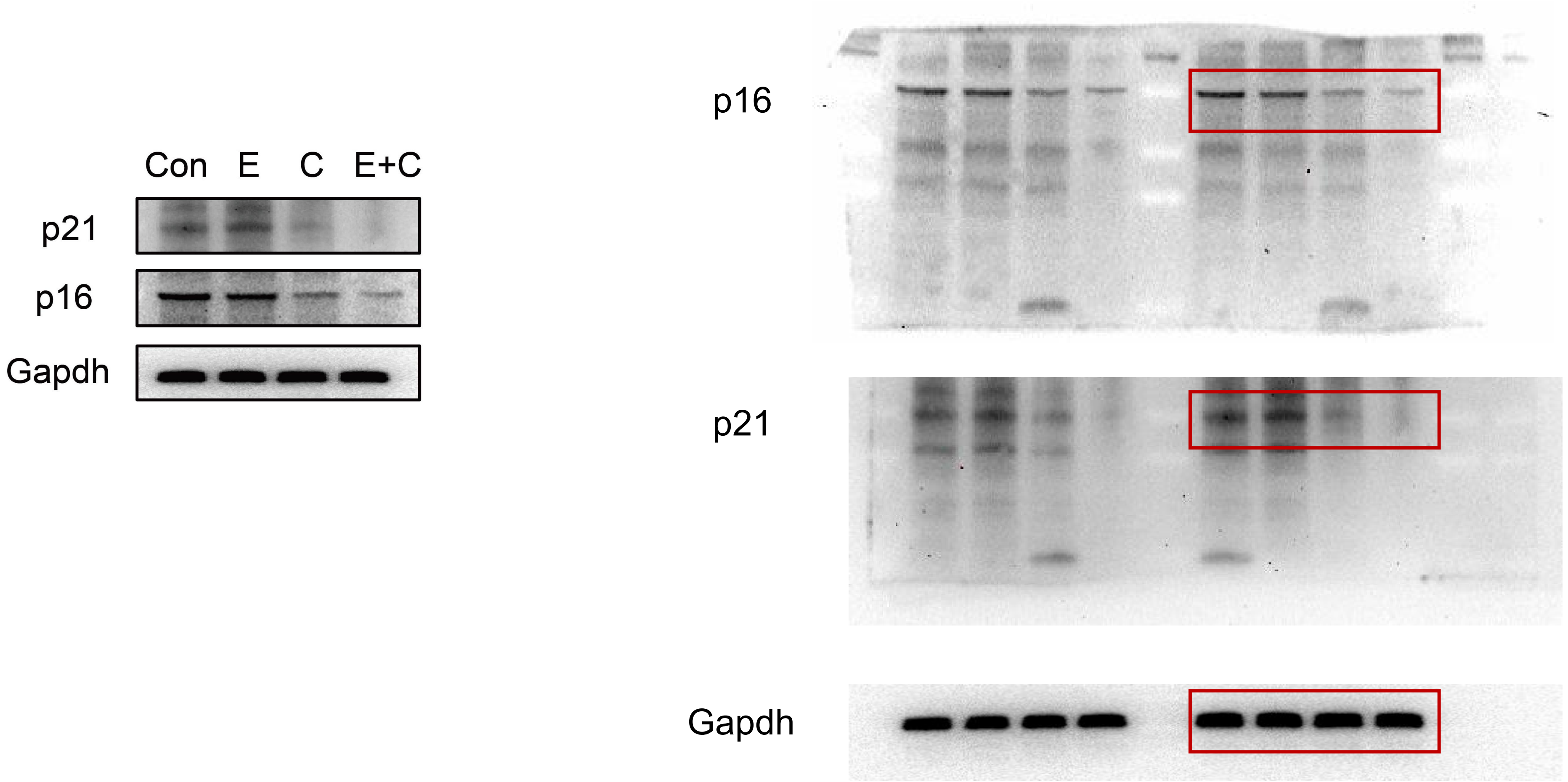

Figure S1

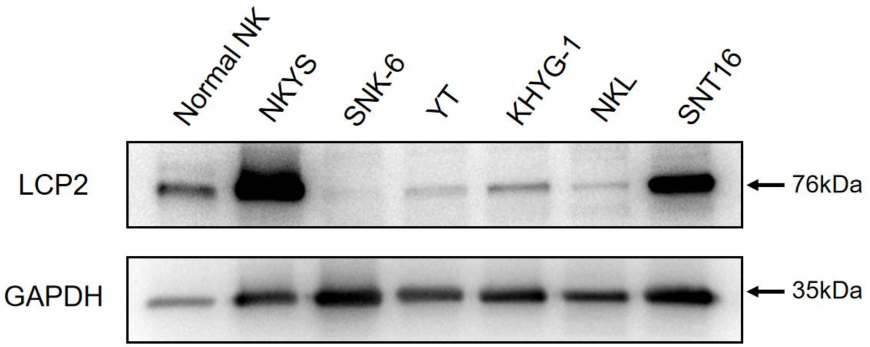
Normal NK
NKYS
SNK-6
YT
KHYG-1
NKL
SNT16
LCP2
GAPDH
76kDa
35kDa

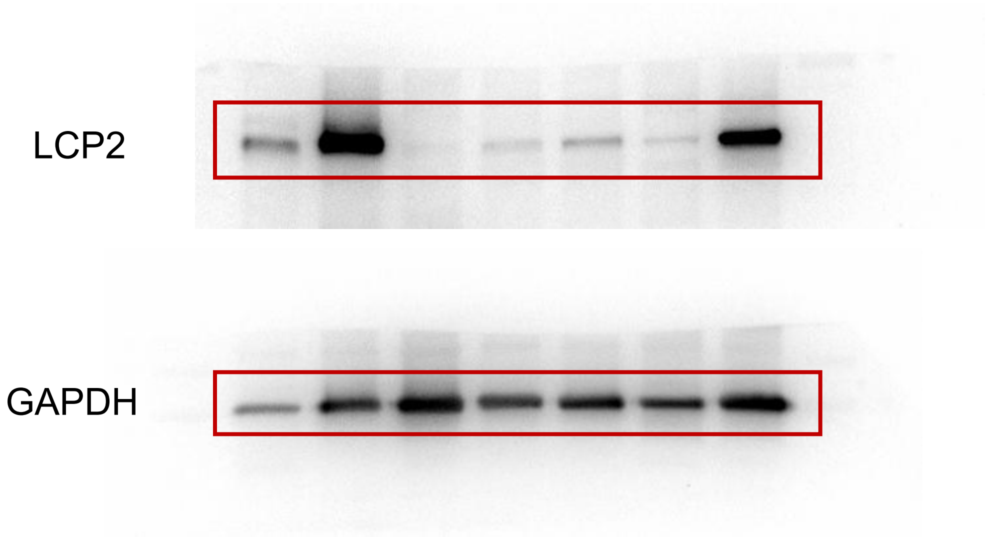
LCP2
GAPDH

Figure S2A

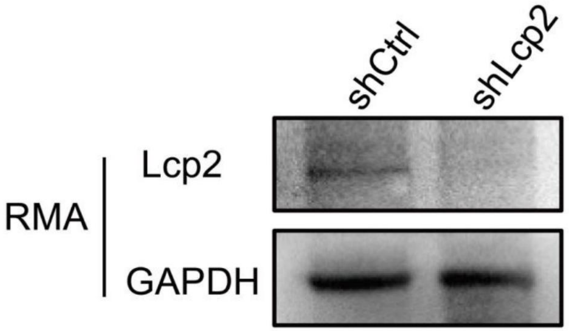

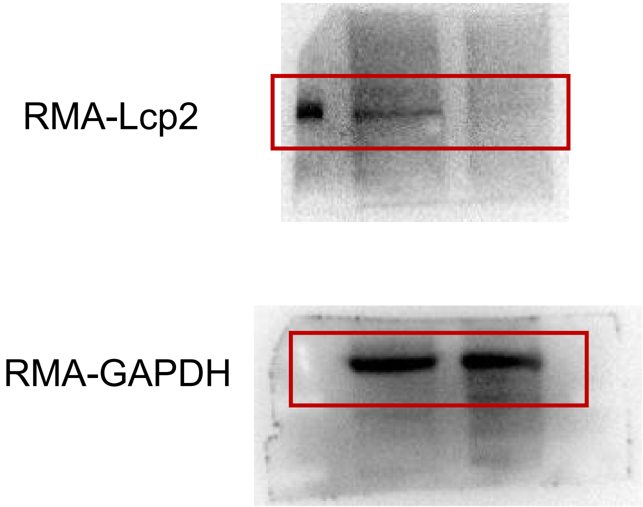

Figure S3A

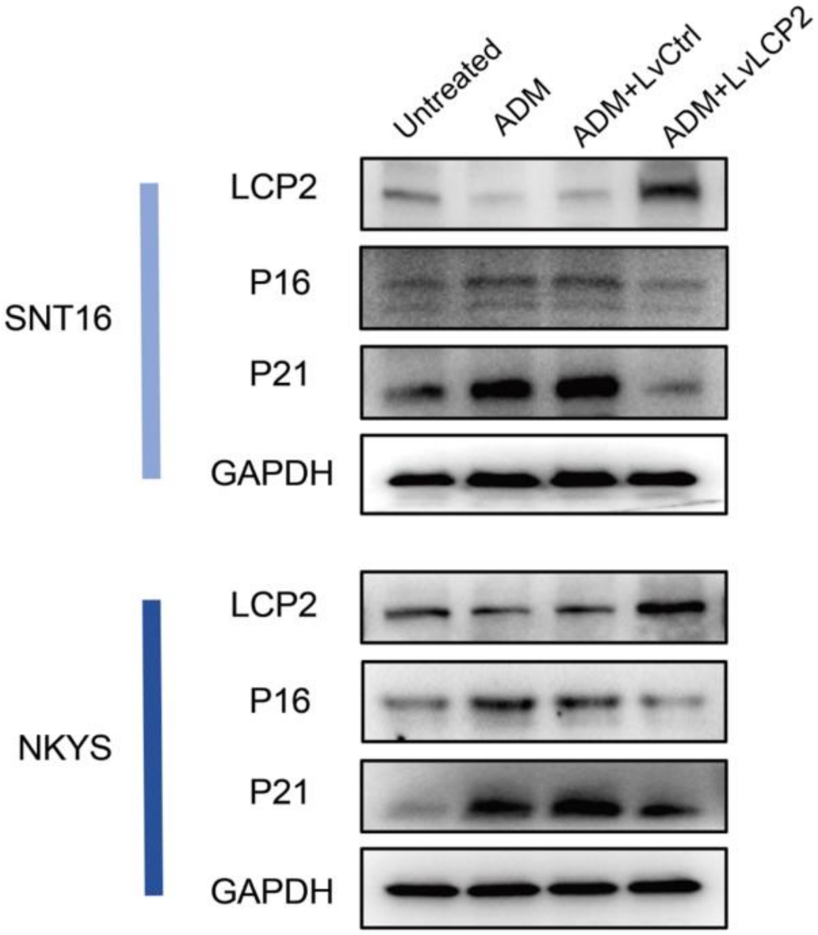

Untreated
ADM
ADM+LvCtrl
ADM+LvLCP2
SNT16
LCP2
P16
P21
GAPDH
NKYS
LCP2
P16
P21
GAPDH

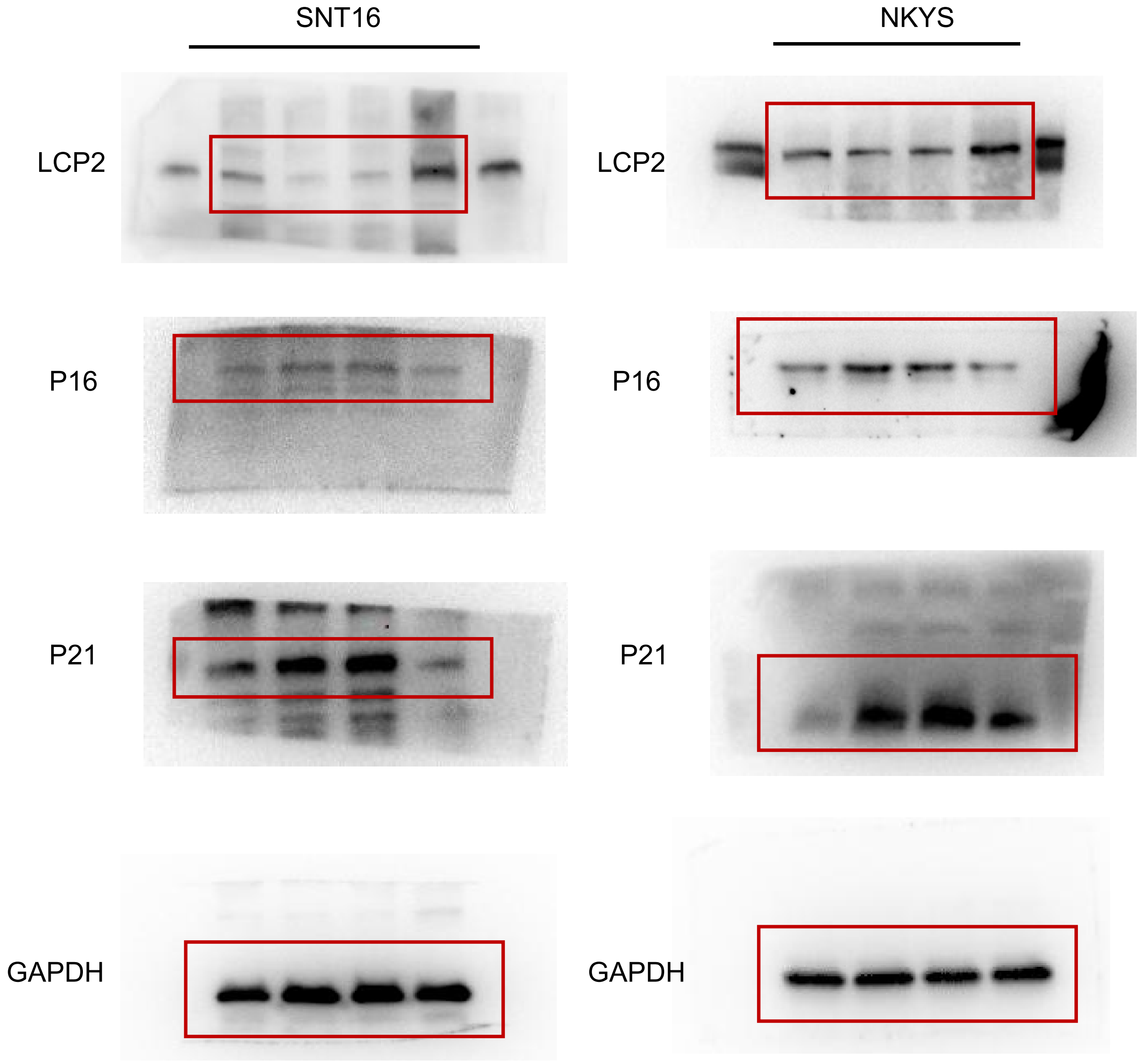

SNT16
NKYS
LCP2
P16
P21
GAPDH
LCP2
P16
P21
GAPDH

Figure S5A

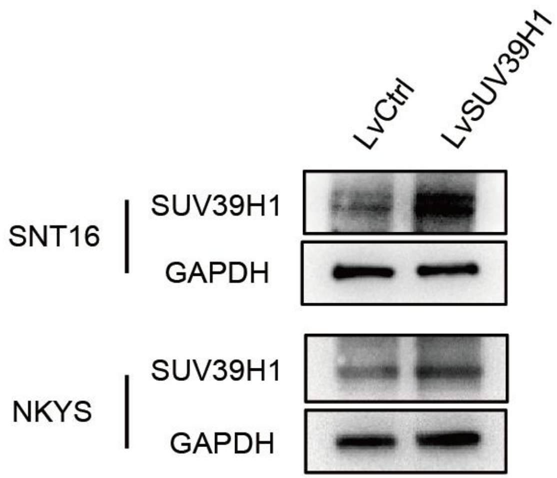
LvCtrl
LvSUV39H1
SNT16
SUV39H1
GAPDH
NKYS
SUV39H1
GAPDH

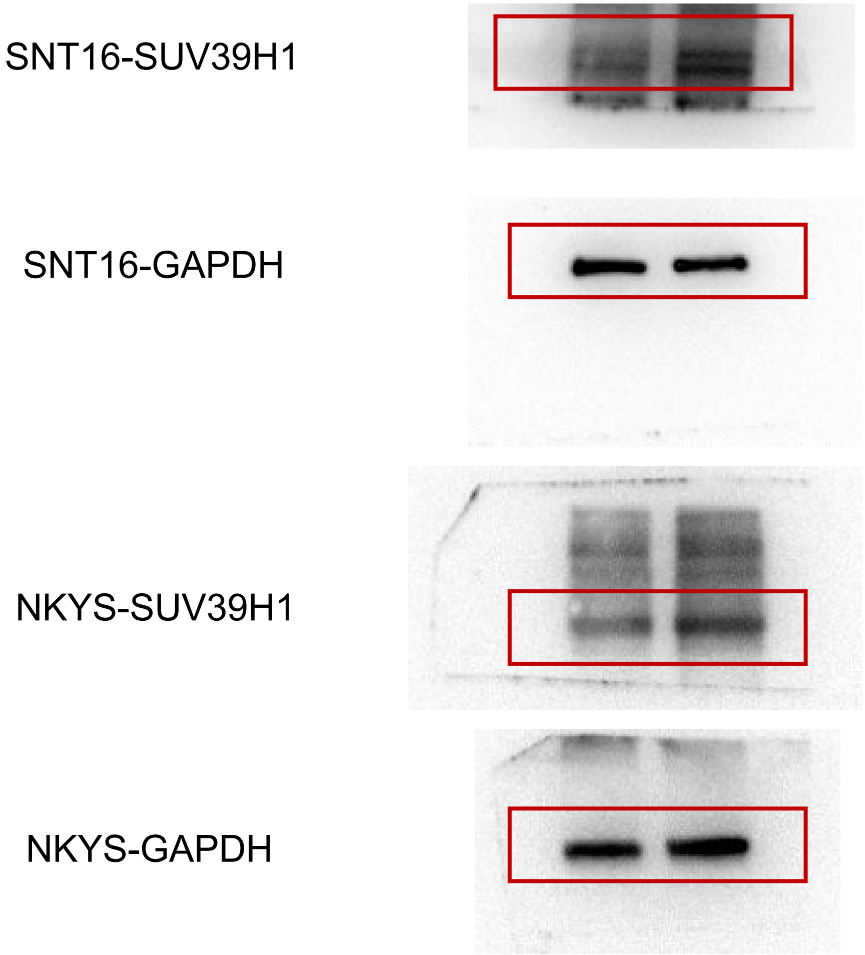
SNT16-SUV39H1
SNT16-GAPDH
NKYS-SUV39H1
NKYS-GAPDH
